# Hazardous materials facility siting optimization and ranking: A transportation risk mitigation framework

**DOI:** 10.1371/journal.pone.0290723

**Published:** 2023-11-15

**Authors:** Musharraf Ahmad Khan, Babak Mehran

**Affiliations:** Urban Mobility and Transportation Informatics Group (UMTIG), Department of Civil Engineering, University of Manitoba, Winnipeg, Manitoba, Canada; National Kaohsiung University of Science and Technology / Industrial University of Ho Chi Minh, TAIWAN

## Abstract

Hazardous material transportation problems have widely been studied in the past especially in the context of routing, scheduling, and network design problems. Yet, the combined hazardous material facility location-routing problem has not been studied adequately. We emphasize that locating a hazardous material facility is a rich process, and a good site can mitigate the potential transportation risk beforehand. A methodological framework is proposed which allows evaluation and ranking of potential sites based on hierarchical relationship utilities. The proposed method attempts to improve the risk functions and applies a stochastic analysis to measure the risk, which relaxes some assumptions in deterministic analysis, and is more realistic while avoiding overestimation of the risk. The study covers multi-objective optimization considering the decision-makers’ preferences on network segments and risk to the population and water bodies. Potential hazardous material facility sites’ rank is determined by the probability of optimality and one-to-one relationship utilities with the points of interests. Results show that the proposed stochastic analysis offers more flexibility to select and rank the potential sites.

## Introduction

### Background and motivations

This research aims to develop a methodology to evaluate and rank potential hazardous material (HAZMAT) facility sites based on transportation risks to the surrounding environment [1]. The Canadian Environmental Protection Act (CEPA), 1999 defines a dangerous good or a hazardous material as a material with properties of flammability, corrosiveness, or inherent toxicity. Transportation of Dangerous Goods Regulations (TDGR) [2], Canada classifies HAZMAT into nine classes which includes 1) Explosives, 2) Gases, 3) Flammable liquids, 4) Flammable solids, 5) Oxidizing substances and organic peroxides, 6) Toxic and infectious substances, 7) Radioactive materials, 8) Corrosives, and 9) Other miscellaneous products, substances, or organisms. Transportation of HAZMAT from one facility to the other is a frequent endeavor as part of everyday economic activities, particularly in the energy sector. The frequent transportation of HAZMAT between different *origin-destination (OD) pairs* becomes complex for two inter-related reasons; first, the several types of HAZMAT to deal with; second, the uncertainties involved during their transportation. Different types of HAZMAT have specific transportation requirements such as vehicle size, loading limits, packaging and safety conditions based on their possible hazardous impact on the surroundings in case of an accident. The uncertainty that an event may happen during HAZMAT transportation and potentially pose adverse effects on the surrounding *assets* such as population and environment has inspired a wide range of research work in recent decades.

**Fig 1:**
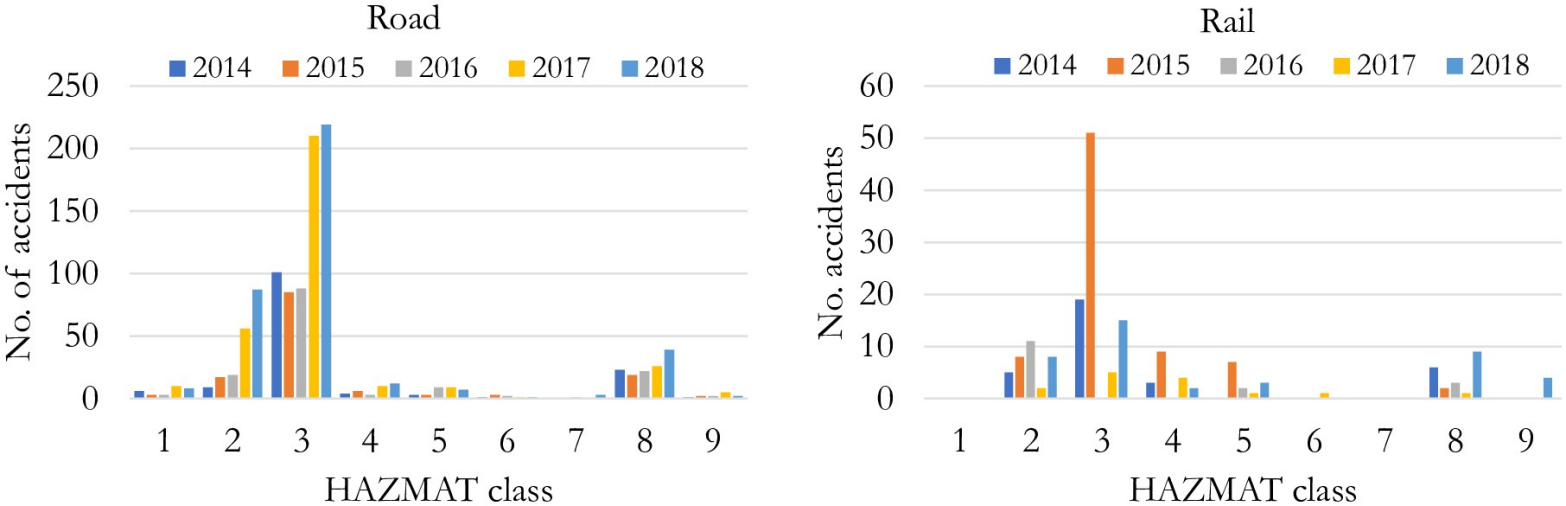
Number of accidents per HAZMAT class in Canada. The chart generated using the data retrieved from [4] and contains information licensed under the Statistics Canada Open Licence, doi:10.25318/3810025601-eng.

**Fig 2:**
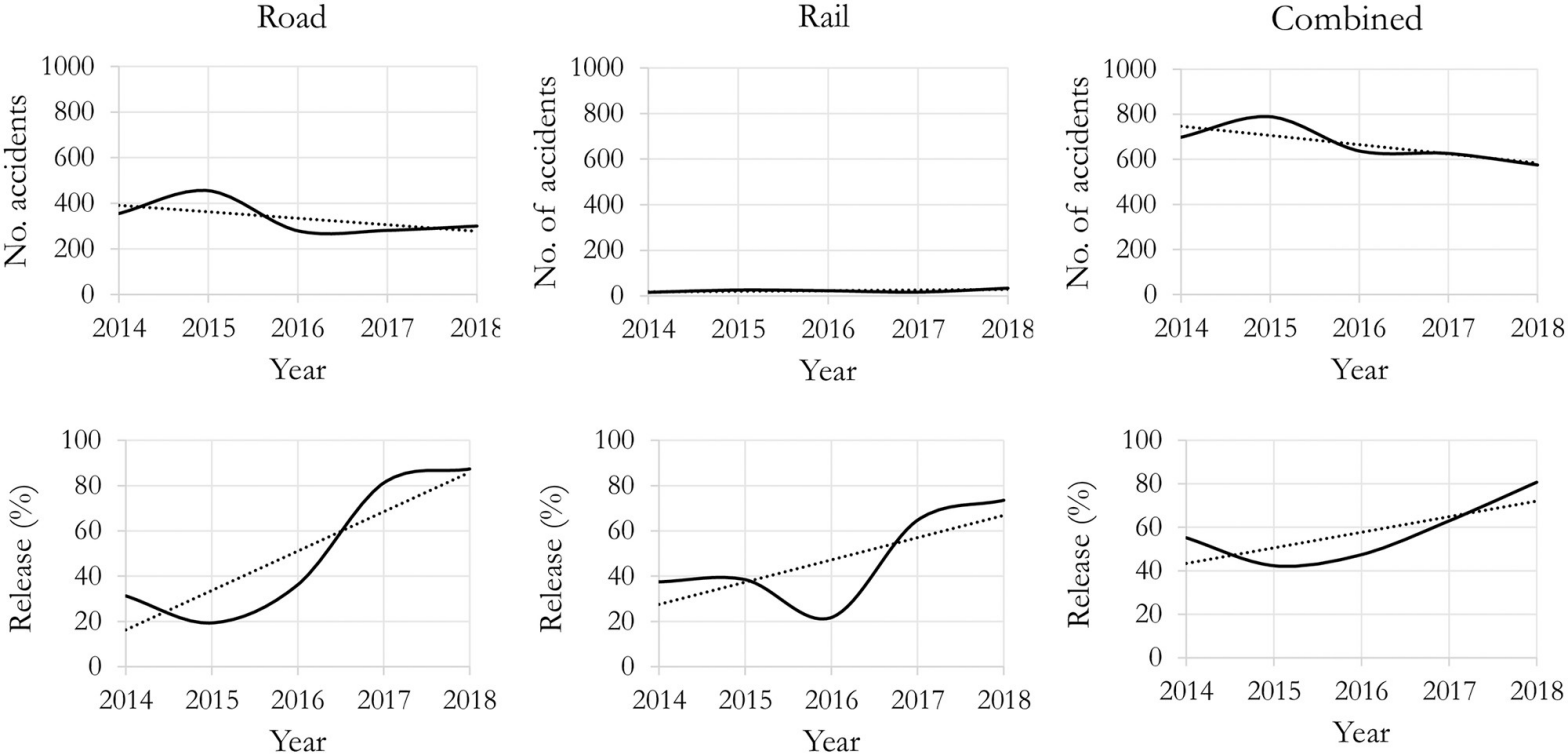
HAZMAT accidents and releases in recent years in Canada. The chart is generated using the data retrieved from [3] and contains information licensed under the Statistics Canada Open Licence, doi:10.25318/3810025201-eng.

There were, on average, 665 HAZMAT involved reportable and non-reportable accidents per year in Canada from 2014 to 2018 [3]. Fig 1 shows the number of accidents for each HAZMAT class for roads and railways [4]. While the overall number of accidents involving HAZMAT in Canada is generally decreasing, the proportion of accidents with total reportable releases of HAZMAT shows increasing trends (Fig 2), which is alarming. HAZMAT involved accidents are often considered High Impact Low Probability (HILP) events, i.e., rare events that can be disastrous, causing loss of lives and significant damage to the environment and assets. For example, the rail disaster occurred in Lac-Megantic in the province of Quebec, Canada, in July 2013, when a 72-car freight train carrying crude oil derailed, resulting in fire and explosion of multiple tank cars. The incident took more than forty-two lives and destroyed more than 30 surrounding buildings [5].

A single incident involving hazardous material can potentially jeopardize the safety of the surrounding population and other assets. It can lead to severe or fatal consequences with substantial costs and other implications such as delays in providing services. The incident can also lead to a domino effect if other HAZMATs or sensitive facilities are in the incident location’s vicinity. Furthermore, the transportation of a HAZMAT from the *points of interest (POIs)*, i.e., the demand and supply point, is interrelated to a HAZMAT facility location. HAZMAT facility location impacts the number of shipments to and from the facility, considering the cost, risk, and time. The increase in HAZMAT shipments’ frequency increases the likelihood of risk exposure; therefore, locating a HAZMAT facility should be a thoughtful process involving thorough analyses of influencing factors.

In this study, we follow a risk mitigation strategy for HAZMAT transportation and propose a methodological framework that allows ranking of the potential HAZMAT facility sites based on i) deterministic as well as stochastic analysis, ii) evaluation of the routes, and iii) multi-objective optimization. Decision-maker’s specified interactive hierarchical-relationship between potential sites and designated POIs are considered. While demonstrating a specific analysis, we propose improved risk functions that are more consistent with the formal definition of risk. In addition to assets exposed, unlike traditional risk functions, essential factors that can affect the resulting consequences, such as the magnitude of the hitting hazard, assets’ vulnerability, and hazard exposure time, are considered in the proposed risk functions by following a relative measure-based approach. We show that the stochastic analysis for risk measurement relaxes few assumptions made in the deterministic analysis and provides a more realistic assessment of transportation risks. We finally demonstrate that the proposed stochastic approach to measure transportation risks is much more practical while providing flexibility in choosing optimal HAZMAT sites considering uncertainties involved in the process.

### Literature review

The LRP is classified by [6] into ‘problem perspective’ and the ‘solution methods used’. Problem perspective includes the nature of demand and supply, i.e., deterministic, and stochastic; the number of facilities, i.e., single or multiple facilities; vehicle fleet size; facility capacity; time windows; and single or multi-objective. Solution methods include exact algorithms and heuristics [7]. categorized the HAZMAT transportation problems into four significant classes by, i.e., routing problem, combined location-routing problem (LRP), network design problem, and risk assessment. Routing involves finding routes between OD pairs, either the best or a combination. It also includes routing and scheduling problems. A combination of routing and facility location addresses the route finding and the facility location as part of the same problem. Network design seeks a sub-network from the leading network suitable for HAZMAT transportation. Lastly, risk assessment addresses the quantification of the risk involved due to HAZMAT transport on routes. When considering location sorting in the context of HAZMAT transportation, LRP can be classified into five categories [8], i.e., origin location (with only outgoing shipments), destination location (with only incoming shipments), processing location (with incoming as well as outgoing shipments), gateway location (that has to be visited with the sole purpose of global risk optimization), and location for an emergency response unit. The readers are suggested to review [9] for a recent and detailed classification of LRP.

In principle, LRP involves two fundamental decisions made interdependently: decision on the location of facilities and decision on the routing of vehicles [10]. The routing component of a standard LRP takes tour planning aspects into account. Thus, the aim is to solve a facility location problem (the “master problem”), but in order to achieve this, the vehicle routing problem (the “subproblem”) needs to be solved simultaneously [11]. Yet, with recent advancements in computational methods and recourses, LRP is being considered a viable and comprehensive approach for the combined optimization of HAZMAT routing and location sorting. [12] explain the concepts, models, methods, and research gaps in LRP. LRP is different from the location-allocation problem (LAP) in that it requires explicit route planning. The LAP ignores explicit route planning while locating the facilities, leading to additional on-ground distribution costs [13].

[14] proposed a multi-objective model to solve LRP for HAZMAT and proposed a heuristic to solve [15]. also proposed a multi-objective incapacitated facility location problem using the classical Ɛ-constraint method [16]. addressed a reliable LRP and proposed a scenario-based mixed-integer programming model to optimize depot location along with delivery routing while considering the disruption risk of the depots. [17] proposed a mathematical model to explicitly consider the equity, however, unlike [18], they used the Gini coefficient to achieve risk equity. Two equity constraints concerning exposure from the network links and HAZMAT sites are introduced to guarantee that the equity goal does not simply generate more hazmat exposure across the entire community. The model is optimized using modified mixed-integer programming for a given hazmat facility location and routing [19]. conducted a comparative study to investigate whether location and routing can be considered separately for solving LRP and concluded that taking location and routing separately or simultaneously doesn’t change the results. However, they recommended a separate routing and location model because of its simplicity and less computational complexity. Since LRP can be computationally challenging, especially when more rigorous analyses considering edge or vertex level analyses are conducted, much literature is published on methods and heuristics to solve LRP efficiently. For instance [20], applied a genetic algorithm (GA) to a multi-objective LRP for HAZMAT producing facilities. Similarly [21], adapted simulated annealing (SA) to an integrated solid waste LRP to solve the problem efficiently [22]. considered fuzziness in transportation costs and the number of people affected in solving LRP for HAZMAT. They applied ‘credibilistic’ chance-constrained programming and proposed a GA to efficiently solve the problem [23]. proposed a model for designing a reliable HAZMAT transportation network based on hub location topology under uncertainties created by HAZMAT incidents and external events [24, 25]. proposed a multi-objective multi-product maximin hazmat LRP considering multiple OD pairs. A mathematical model is proposed with five decision variables, including minimum weighted distance from vulnerable population units to HAZMAT facility, minimum weighted distance to the network edges used for hazmat transportation, the flow of hazmat, quantity of hazmat offered in hazmat facility and exposure hazard of non-vulnerable population units due to hazmat flow along network edges within the danger zone and the amount produced in the facilities whose danger-facility area is the population unit. The method used weights for each objective function and integrated them after making them dimensionless. This approach is a classic example of combining the objectives and solving the combined objective function (see also [26]). However, this approach is not an ideal approach for a multi-objective optimization problem [27]. proposed a multi-objective LRP model for HAZMAT logistics with traffic restriction constraints and proposed two GA algorithms to solve [28]. attempted an integrated approach for routing and location problems for HAZMAT in a bi-modal transportation network by applying maximum likelihood sampling and Sample Average Approximation [29], proposing a new hybrid heuristic for multiple scenarios application. New methods have also been proposed in the context of HAZMAT transportation to solve LRP, such as by [30], who proposed a robust possibilistic programming model considering the risk of transportation to population. However, they recommend usage of efficient heuristics and novel meta heuristics to solve LRP optimization problem, as recommended by others such as [31–33]. It can be seen that the majority of the LRP literature presents deterministic models for solution method [6]. mentioned the need of stochastic models and stressed upon to build conceptual foundations for stochastic analysis as many of the variables involved in a generic LRP are random variables such as customer locations, travel time, etc. [34] proposed a multi-objective stochastic mixed-integer nonlinear programming model that was solved using non-dominated sorting genetic algorithm-II (NSGA-II) and Monte Carlo simulation to overcome the stochastic combinatorial optimization problem of the study [28]. attempted an integrated approach for routing and location problems for HAZMAT in a bi-modal transportation network by applying maximum likelihood sampling and Sample Average Approximation [29], proposing a new hybrid heuristic for multiple scenarios application. tackled these uncertainties in hub-transportation networks and proposed a bi-objective reliable capacitated *p*-hub location model to minimize transportation cost and time.

### Contribution

The LRP in the HAZMAT context has not been widely studied in the last twenty-five years, see [8, 35]. It is until very recently that the researchers are more interested in the intricate nature of the problem. This study addresses a variant of the LRP problem, which *ranks* the sites as part of the location problem, which is a significant advantage and has not been explicitly attempted before up to the authors’ knowledge. Ranking of the sites has an added advantage especially when the optimal site is not practically feasible due to some unavoidable constraint. It also provides flexibility in selecting an optimized site when multi-disciplinary location preferences need to be addressed.

Risk estimation is foundational in HAZMAT related problems. In literature, the risk has primarily been measured either in terms of the accident probability on route links or a combination of probability and consequence, where the consequence is in terms of population affected. There are other elements related to the consequence, such as community resilience or preparedness against the hazard, hazard intensity and exposure time, which have been rarely found in the literature, perhaps due to the complexities involved in their calculations. In the traditional risk estimation approach, three basic assumptions prevails in the literature to model the transportation risk, the uniform incident probability of a route edge throughout its reach, the uniform density of the assets around a route edge, and all assets are exposed to the hazard no matter where the incident has happened along the edge. The first assumption doesn’t hold if the distribution of incidents on an edge is not uniformly distributed, which is quite a practical case, for example, the number of incidents near the intersections is greater as compared to the middle section of the edge. The third assumption provides a tool to analyze the maximum-risk scenario along the route; however, this can overestimate the risk unnecessarily since the accident will only happen at a certain location on the edge. These three approaches entail from the deterministic risk estimation approach in which a continuous buffer is considered along the network segments as an influential area to calculate the risk. It is very recently that researchers are valuing the stochastic framework to address this complicated problem. For example [35], mentions that to model population distribution that changes over time, a stochastic framework would more likely be required, indicating that the stochastic models align with the future direction in HAZMAT problems. The current study is unique in proposing the stochastic analysis on risk which is not attempted before. Also, the proposed risk estimation is incident-based along with the distance-based, which makes it distinctive. To this account, the traditional risk functions are enhanced in the current study to support the stochastic risk estimation which relatively provide accurate risk estimation than the traditional deterministic analysis.

In addition, solving LRP in a multi-modal setting is rarely attempted. This study considers a multi-modal network in a multi-objective setting and is unique in incorporating decision-makers’ preferences in the problem.

Table 1 presents the standing of the current work in the comprehensive overview of LRP literature from the last 25 years incorporating recent review by [8]. The next section describes the proposed stochastic methodology and the methodological framework in detail. The application and results section describes the demonstration of the proposed methodology for a real-world application, followed by the conclusion and future research directions.

**Table 1:** Summary of key literature on LRP (last 25 years).

Reference	Risk			Coupling criteria	Optimization Concept	Network type	Location type	Locationranking
	Type	Estimation approach	Impact basis					
[36]	PE	-	LB, EPB	CT	PO	SM	DL	-
[37]	PE	-	LDB	DU, CT	GP Trade-off	TN	DL	-
[38]	AP, PE	-	-	CT	-	TN	PL, DL	-
[39]	AP, PE	-	-	CT	GP trade-off	TN	PL, DL	-
[40]	AP, PE	-	-	CT	-	-	PL, DL	-
[41]	PE	D	SB	CT	MIP Trade-off	-	PL, DL	-
[42]	PE	D	SB	CT	-	SM	PL, DL	-
[43]	AP	-	-	DT	-	SM	DL	-
[44]	PE	D	SB	CT	PO	SM	DL	-
[45]	PE	D	SB	CT	PO	SM	GL	PR
[46]	PE	D	SB	CT	PO	SM	GL	-
[47]	ENV	D	SB	CT	PO	SM	PL, DL	-
[48]	AP, PE	F	RV	TT	PO	SM	OL	-
[16]	FL	D, S(FL)	RV	CT	MIP Trade-off	SM	OL	-
[49]	ENV	D, S(CT)	RV	CT	PO	SM	PL, DL	-
[17]	AR	-	-	DT	Trade-off	SM	DL, PL	-
[50]	PE, ENV	D	SB	CT	PO	SM	PL, DL	-
[23]	AP, PE, TY	D, S(TY)	LDB, EPB	CT, TT	PO	MM	TL	-
[28]	PE, TY	D, S(TY)	SB	CT	PO	MM	TL	-
**Current work**	**PE, ENV**	**D, S(PE, ENV)**	**SB, DB, IB**	**PF**	**PO**	**MM**	**PL**	**FR**

Risk type: AP (Accident probability), PE (Population exposure), ENV (Environment), TY (Terminal yards), FL (Facilities), AR (Accident rate); Risk estimation approach: D (Deterministic), S(c) (Stochastic on criterion *c*); Impact measurement basis: SB (Segment buffer), IB (Incidence location buffer), DB (Distance-only based), LDB (HAZMAT Load-Distance based), EPB (Estimate population based (from external sources)), RV (Random value); Coupling criteria: TT (Travel time), CT (Cost), DT (Distance), DU (Disutility (HAZMAT facility size and approaching distance)), PF (Decision makers’ Preference on segment categories); Optimization concept: PO (Pareto-optimality), MIP Trade-off (Mixed-Integer Programming Trade-off), GP Trade-off (Goal Programming Trade-off); Network type: MM (Multi-modal), SM (Single modal), TN (Tested network); Location type: DL (Destinations (incoming only)), PL (Processing locations (incoming and outgoing)), OL (Origins (outgoing only)), TL (Transfer or Hub locations), GL (Gateway locations); Zone ranking: PR (Partial ranking (ranking single solution with respect to risk type)), FR (Full ranking (multiple solutions with ranks))

## Methodology

### Site evaluation concept

The importance of a HAZMAT facility’s location can be characterized by the number of shipments it can produce or serve under a specified condition. These shipments can originate from the HAZMAT facility itself or any supply location or POI(s). There can be multiple numbers of POIs associated with a hazmat facility, and the geospatial relationships can determine the hazmat facility’s significance with respect to a specific POI or all POIs collectively. A *relationship* represents the quality interaction between a POI and a potential HAZMAT facility site, also called a *zone* in the later sections of the article. The individual relationship, also called the *one-to-one relationship*, represents the POI’s importance to the hazmat facility. The relationship on a collective basis, called the *collective relationship*, signifies the facility location’s suitability in conjunction with all POIs. These relationships can take many attributes depending upon the *evaluation criteria* selected by the decision-maker. Examples may include but are not limited to distance, speed, time, or even safety or security or a combination of these using a relationship function.

**Fig 3:**
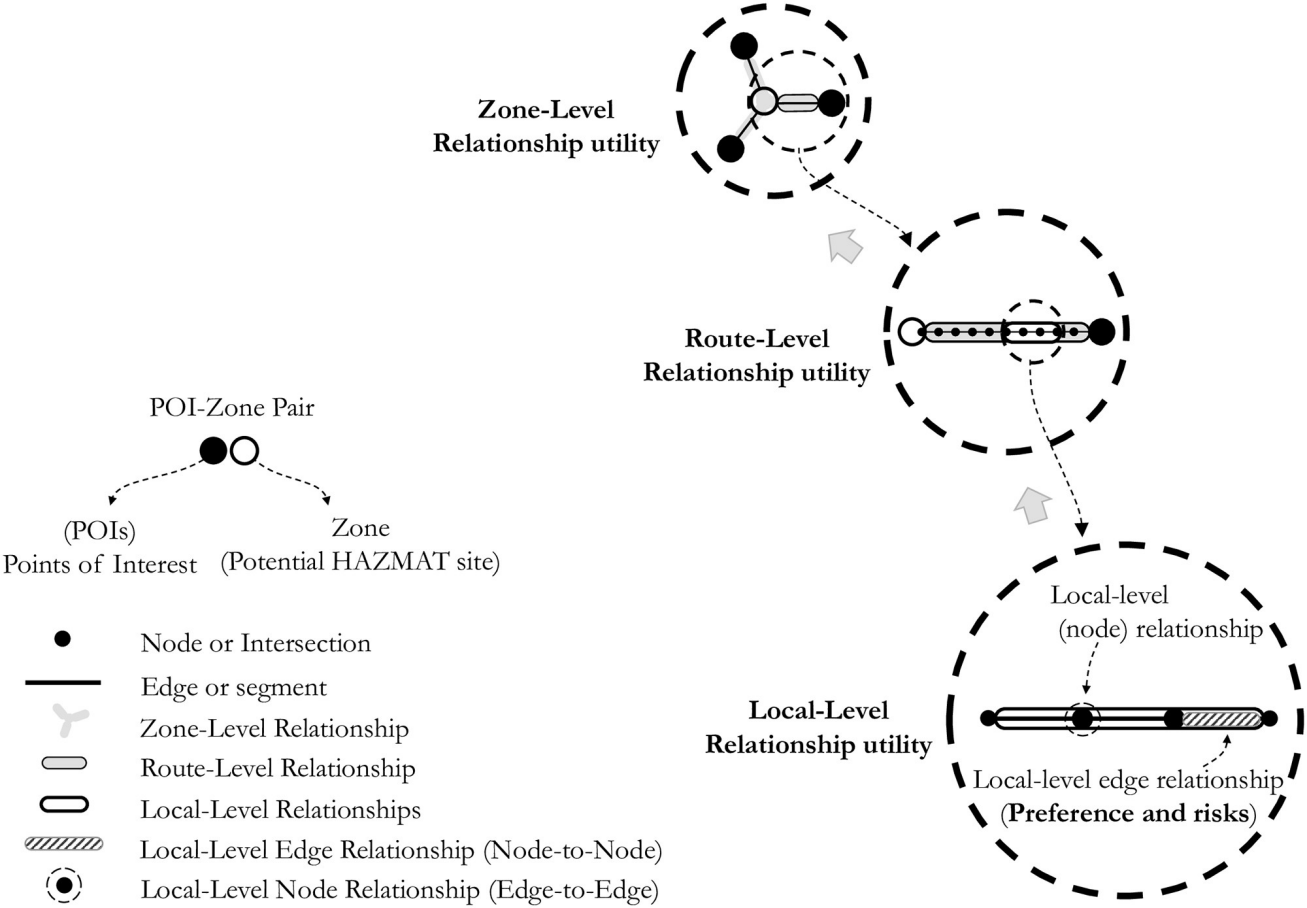
Description of hierarchical relationships.

The individual relationship (between a POI and a potential site or a zone) is comprised of small local level relationship components, i.e., node-to-node (edge or link) level or edge-to-edge (node or intersection) level, which form a chain called a *path*. Thus, there can be three levels of relationships between the set of POIs and the potential sites within a network, i.e., *Local-Level relationship*, *Route-Level relationship*, and *Zone-Level relationship*. The zone-level relationship can be measured by combining the route-level relationships from all POIs for a particular zone. Fig 3 presents the graphical view of this concept. Table 2 defines the key concept elements and notations.

**Table 2:** Essential elements of the base concept.

Notation	Element	Definition	Description
***C***	Evaluation Criteria	A set of evaluation criteria.	A set of all evaluation criteria to evaluate the zones which expresses the interactive relationship between an OD pair.
***c***	A criterion	Evaluation criterion	A chosen criterion from a set of all evaluation criteria *C* by the decision-maker to evaluate the zones which expresses the interactive relationship between an OD pair.
***e***	Local element	A smallest network element under consideration.	A segment (link or an edge) or an intersection (node) in the network.
***f*(*e***_***c***_ **)**	Relationship function	A mathematical function in terms of criterion *c* for a local element *e*.	A mathematical expression developed to quantify relationship utility for a local element of the network considering an evaluation criterion. A representation of the interactive relationship between a POI and a zone. This interaction is characterized by the selected criteria and assigned as attribute to the local elements.
***δ***	Local-level utility	A quantified value of the relationship utility of local element based on the relationship function considering a criterion.	${\delta_{\;e}^{c}}_{\;}$ is the relationship utility for local element *e* based on the relationship function developed considering criterion *c*.
**Ф**	A route	A path connecting a POI-Zone pair.	A route is a combination of *n* local elements (segments and intersections) starting from a POI and ending at a zone or vice versa, without repeating any intermediate node and edge. ${Ф}_{oz\;}^{c}$ is the route from POI *o* to zone *z* considering criterion *c*.
***φ***	Best route	A path with maximum route-level utility (depending upon the criteria and objective), between a POI and a zone.	*φ*_*oz*_ is the best route between a POI *o* and a zone *z*. Best route can also be called the shortest route if the maximum local-level utility seek out for each local element of the route is represented by the minimization of the objective function or relationship function.
U	Segments of the best route	A set of all segments in a best route *φ*.	If the best route from POI *o* to zone *z* based on criteria *c* is represented by $\varphi_{oz}^{c}$, then U represents the set of all segments of the route $\varphi_{oz}^{c}$. The set U corresponds to the route $\varphi_{oz}^{c}$ or $U≙\varphi_{oz}^{c}$.
***ζ***	Route-level utility	$\sum_{e=1}^{n}{\delta_{e\;}^{c}}_{\;}^{\;}$, where *n* is the total local elements in the route Ф based on criterion *c*.	Relationship utility at route level between a POI and a zone, based on criterion *c*. $\zeta_{\varphi_{oz}^{r}}^{c}$ is the route-level utility based on criterion *c*, developed upon the best route between a POI *o* and a zone *z* considering some criterion *r* such that {*r*, *c*} ∈ *C*.
***Ω***	Zone-level utility	$\sum_{Ф=1}^{m}\;\zeta_{Ф}^{c}$, where *m* is the total routes from a zone to all POIs based on criterion *c*.	Relationship utility at zone level based on criterion *c*. *$\Omega_{\Phi_{z}}^{c}$* and $\Omega_{\varphi_{z}}^{c}$ are the zone-level utilities based on ‘any’ route and the ‘best’ route between all POIs and a zone *z* considering criterion *c*, respectively.

As shown in Fig 3, a hierarchical approach is proposed, which starts at the local level, reaches the route level, and finally to the zone level. The development of the *relationship function* forms the basis for relationship quality and should be dealt with care. The quality of the relationship is defined by the *relationship utility* value, which is the output of the mathematical expression describing the relationship function. Risk or safety functions are examples of the relationship function. For instance, if the risk is considered as a criterion, the objective would be to minimize the value of the mathematical risk function representing the relationship utility between a zone and a POI, considering risk function as a *relationship attribute* of the local network elements (segments or intersections). Similarly, if the intention is to set safety as a criterion, the objective would be to seek maximum relationship utility taking safety function as a relationship attribute of the network’s local elements. Once these relationships are quantified as relationship utilities, the significance of the hazmat facility can be measured quantitatively.

### Methodological framework

**Fig 4:**
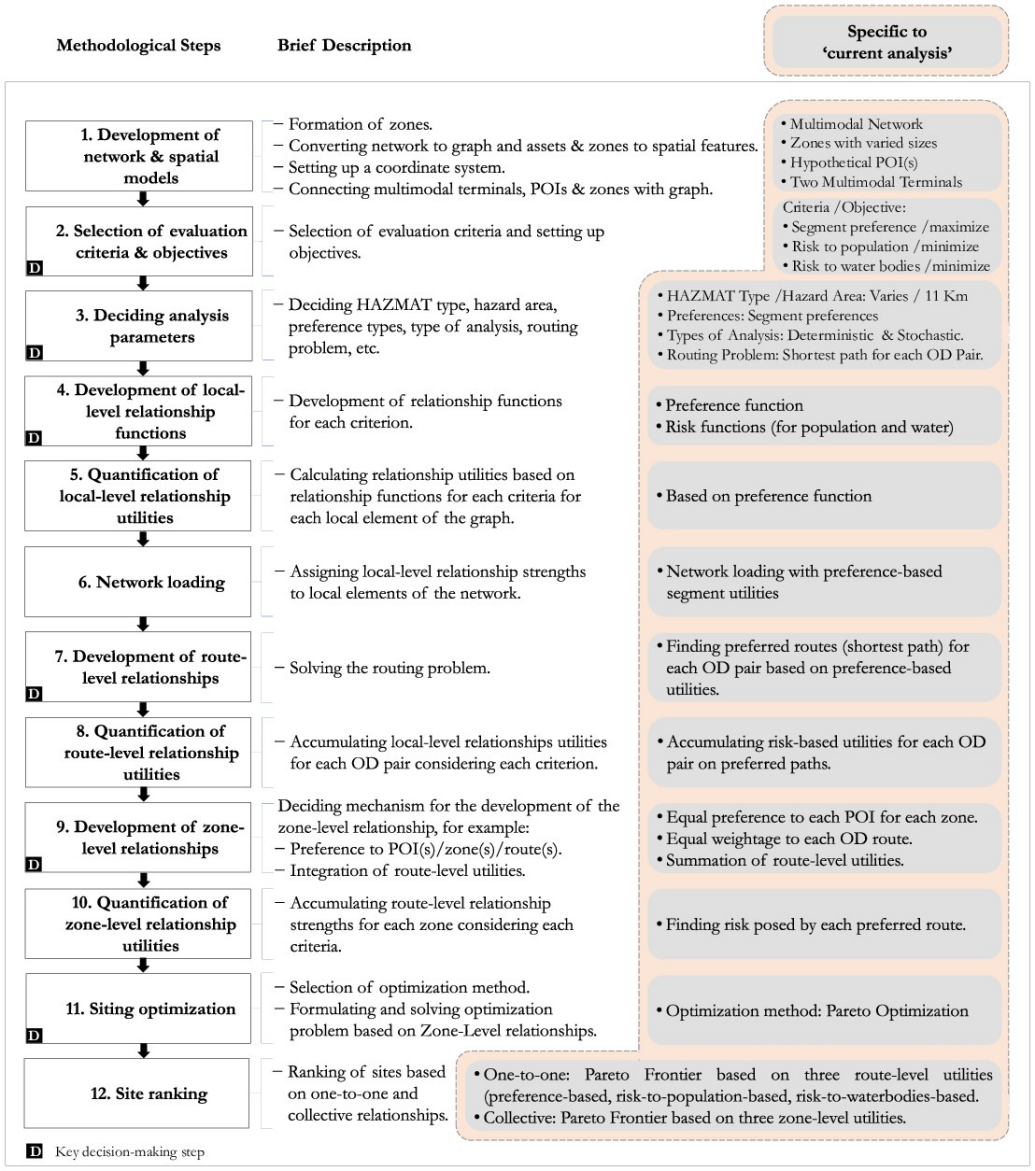
Proposed methodological framework.

We propose a methodological framework comprised of twelve steps to evaluate and rank the potential HAZMAT facility sites (Fig 4). *Step* 1 is the development of network and spatial models and requires information such as specifying POIs, coordinate system, accessing road parameters, zoning, and forming a multi-modal network graph ready for analysis. There are six key *decision-making* steps in the proposed framework; *Step* 2 is the selection of evaluation criteria and their respective objectives, which helps to formulate the optimization problem; *Step* 3 is the decision on HAZMAT type, which enables setting the *hazard area*, i.e. the area within which the assets are subject to the risk; type of decision-makers’ preferences to be incorporated into the methodology and their quantitative values; the routing problem to be solved; and the type of analysis to be performed; *Step* 4 is the decision on relationship-functions such as risk functions; iv) *Step* 7 is the decision on the algorithm or heuristic to solve the routing problem; *Step* 9 is the decision on the mechanism to develop a zone-level relationship; and *Step* 11 is the selection of optimization method and heuristics to solve the siting optimization problem. *Steps* 5, 6, 8 and 10 are the quantification steps. *Step* 12 related to usage of site optimization results to rank the sites. The following sections present the details of these methodological steps and demonstrate a specific analysis referred to as ‘current analysis’ in the rest of the article.

### Development of network and spatial models

The first step is the development of the network model. The network model development includes the formation of a multimodal network as a network graph. Converting the network into a graph enables us to analyze the network using graph theory. However, to make the required analyses possible, it is necessary to connect all the elements within the graph. In a multimodal network, multimodal terminals play the role of designated transfer points for the freight between different types of networks, such as rail to road and vice versa. In the current analysis, multimodal terminals are represented as designated nodes in the graph.

Similarly, POIs are also represented as designated nodes in the graph. The formation of potential zones divides the whole study area into smaller zones that could be considered potential sites for a hazmat facility. These zones connect to the graph via their point of representation, generally taken as the geometric centroid. The segment and intersection attributes should be loaded to the edges and nodes of the graph, respectively. Table 3 describes the essential elements of the network model.

**Table 3:** Essential elements of the network model.

Notation	Element	Definition	Description
***G***	Multimodal transportation network model	A directed Graph.	A mathematical model of a network comprised of the railway and hierarchical road network, coded as a directed graph (segments have directions).
***S***	Segments	A set of all edges in *G*. One edge (segment) is represented by *s* such that *s* ∈ *S*.	An edge is a representation of a continuous road or railway link or section in the network which starts and ends at specific locations such as intersections or cul-de-sacs, having a certain length.
***X***	Intersections	A set of all nodes in *G*. One node (intersection) is represented by *x* such that *x* ∈ *X*.	A node is the representation of the endpoint of an edge (road or railway segments) either located alone or connecting two or more edges forming a junction.
***T***	Multimodal terminals	Set of designated nodes in *G*.	Nodes are considered as transfer points for the freight between transportation modes.
***O***	Designated POIs	Set of designated nodes in *G*.	Nodes connected in *G* and represents potential HAZMAT supply or demand locations. A single POI is represented by *o* such that *o* ∈ *O*. The total number of POIs in set *O* is also represented by *O*, where applicable.
***Z***	Zones	Set of designated nodes in *G*.	Representation of the geometrical centroids of the potential HAZMAT facility sites that need to be evaluated. A single potential zone is represented by *z* such that *z* ∈ *Z*.
**E**	Emergency Response Service Stations	Set of designated nodes in *G*.	Representation of the location of the Emergency Response Service Stations as nodes connected in *G*.

In addition to the network model, a spatial model is also required to allow risk measurement of the surrounding affected assets. The spatial model comprises the spatial region (study area), which encloses the multimodal network and geographical areas such as population centers and the water bodies. In keeping the analysis consistent to produce correct results, it is necessary to set the same projection for the graph and the spatial regions. The coordinate system saves the geographical reference of the spatial elements in the form of numerical coordinates. All the spatial attributes and their coordinates are stored in the attribute tables. Table 4 presents details of some essential elements of the spatial model for the current analysis.

**Table 4:** Essential elements of the spatial model.

Notation	Element	Definition	Description
**Δ**	Population centers	Population polygons located in the same geographical plane as the network, considered as assets.	Representation of the population centers located around the network elements.
**Y**	Waterbodies	Waterbody polygons and lines located in the same geographical plane as the network, considered as assets.	Representation of rivers and lakes
***B***	Hazard Area Limits	A polygon area in the same geographical plane as the network, considered dangerous for the assets.	Representation of the area that can be influenced by a HAZMAT. *B*_*s*_ represents the buffer distance around a segment and *B*_*x*_ represents the radius for a circular hazard area (*hazard circle*) at a particular location *x* on the segment.

### Evaluation criteria and objectives

The selection of the evaluation criteria based on which the sites will be assessed forms the basis for the analyses. The formulation of the relationship function depends on these criteria and associated objectives. For example, the criterion ‘distance’ and the objective ‘to minimize’ can be of interest to the decision-maker; similarly, the criterion of ‘risk to the population’ and the objective ‘to minimize’ the risk function value can be of crucial interest for HAZMAT transportation. For the current analysis, three criteria vitally relevant to the HAZMAT transportation have been considered, i.e., 1) preference of the decision-maker on segment categories (rail/road) for hazmat transportation, 2) potential risk of hazmat transportation to the surrounding population, and 3) the risk to the surrounding surface water bodies. The objectives are to maximize the preference on segment categories and minimize the risks to the population and water bodies while achieving multi-objective optimization. In particular, we intend to develop preferred route-level relationships and want to evaluate the zones based on those preferred relationships and associated risk-based relationships.

### Development of relationship functions

#### Preference function

The decision-makers sometimes make decisions based on their preferences. The possible reason for this could be a lack of concrete data and information, resources, or time. These limitations tend to make the decision-makers inevitably use their personal experience and knowledge to avoid any unwanted scenario or favour any particular aspect. In the current analysis, we have incorporated the decision-makers’ preferences on different network segments, including rail and road categories, for the desired HAZMAT transportation route’s sanity. Generally, experts or decision-makers from various departments participate in a survey to score their preferences from lowest to highest on a designated scale, as described in [26]. Based on these scores, the relationship function for a particular segment can be developed and quantified [26]. propose a preference-based function which can also be expressed in terms of any local element of the network as:

$$\delta_{\;e}^{\theta }={ϴ}_{e}\;\times \vartheta_{e},$$ (1)

Where $\delta_{\;e}^{\theta }$ is the preference-based relationship function for a particular network local element *e*, *ϴ*_*e*_ and *ϑ*_*e*_ are the mean preference score and attribute of the element *e* (e.g., length), respectively.

#### Risk function

As described earlier, researchers interpreted the risk in many ways. However, there is a common consensus to quantify the risk by multiplying the probability of the event’s occurrence by the consequences it causes:

ℝ=ℙ×ℂ, (2)

Where ℙ is the probability that an unfavourable event will occur or the *hazard probability*, and ℂ is the resulting damage or the *Hazard Consequence*. Eq (2) is a straightforward representation and can be exploited based on a particular scenario under consideration, as described in subsequent sections.

*Hazard probability*. The probability of hazard occurrence is the multiplication of sub-probabilities associated with the causal factors contributing to the happening of the event. Calculating this total probability is a challenging task due to the involvement of many causal factors. Decision-makers must always decide which probabilities are most relevant to the problem and can be calculated based on the available information. In the context of HAZMAT transportation, three probabilities are most relevant: the *incident probability*, *release probability*, i.e., the likelihood of the HAZMAT release due to the incident, and the *consequence probability*. Consequence probability is the probability that the HAZMAT, once released, will pose consequences to the surrounding assets. It is part of the hazard consequence. The HAZMAT release is a function of the severity and type of the incident and the HAZMAT package’s soundness. The severity and type of the incident cannot be predicted, although we can judge the package’s soundness through extensive testing before launching the HAZMAT for transportation. Since HAZMAT always tends to pose adverse effects once released, the concern is the quantity of the HAZMAT released, which could negatively affect the surrounding assets. The measurement of this quantity is highly unpredictable since it is again a function of the severity and type of the incident and the kind of rupture that could occur to the package due to package design and material characteristics. We merge the release probability and consequence probability, rationalizing that we are only interested in the adequate amount of HAZMAT release, which has the tendency to affect the surroundings and is called the *effective release*. We call this combined probability a *release and effect probability*. We will explain in the in the coming sections how this probability can be quantified considering stochastic variations. Thus, in a HAZMAT context, we assume that any incident releases the HAZMAT sufficiently to pose some degree of adverse effect on the surrounding assets. Thus, the release and effect probability equals to ‘1’. Eq (2) is revised as follows:

$$\mathbb{R}=p_{c}\;\times \mathbb{C},$$ (3)

Where *p*_*c*_ is the crash or incident probability.

Incident probability on a network segment can be a function of many causal factors such as the number of lanes, Annual Average Daily Traffic (AADT), traffic mix and distribution, percentage of heavy vehicles in traffic flow, capacity constraints, length of the segment, posted speed limits, number and type of intersections, etcetera. Any quantified probability based on these factors is always relative to the context. One may select the level of detail that qualifies the comparison among the network segments. For example, the segment’s length, in the simplest case, can reasonably indicate the tendency towards crashes. It reflects the vehicle’s time on the road, exposing the hazard to the surrounding assets. The longer a HAZMAT is on the road, the longer the exposure time hence the likelihood of an incident. Similarly, crash rates may be a function of the AADT of a segment of a given length. The following model can be assumed to represent the relationships between crash rates and the explanatory variables [51]:

$$\lambda_{\;}=exp\left(\omega \tau \right),$$ (4)

Where *λ* is the crash rate, *ω* and *τ* are vectors of estimable coefficients and explanatory variables, respectively. Eq (4) offers the flexibility to include any explanatory variables (as discussed earlier) the decision-maker wishes to consider depending upon the requirement, significance, or the observed data. According to the Highway Safety Manual (HSM) [52], crash rates can be predicted using Safety Performance Functions (SPF) for different categories of highway segments and collision types. The equation presented below can be used for multiple vehicle non-driveway collisions:

$$\lambda_{\;}=exp\left[\alpha +\beta \times ln\left(AADT\right)+ln\left(l_{\;}\right)\right],$$ (5)

Where *l* is the segment’s length, *α* and *β* are regression coefficients for a particular segment category. Turla et al. (2019) used a similar function for railway segments:

$$\lambda_{\;}=exp\left[\alpha +\beta \times Y+\theta \times M_{\;}\right],$$ (6)

Where *θ* is a traffic exposure coefficient, *Y* is the year index, and *M* is the traffic exposure in billion train-miles.

Based on the predicted crash rates, the Poisson distribution can be used to calculate the incident probability. Since the expected number of crashes is based on annual data, the analysis period is one year. Also, for relative comparison, it is sufficient to consider the probability of only one incident; hence the Poisson distribution takes the form:

$$p_{\;\left(1\right)}=\lambda_{\;}^{{\;}_{\;}}\times exp\left(-\lambda_{\;}\right),$$ (7)

Where *p*_(1)_ is the probability of one incident, and *λ* is the expected crash rate of the segment.

**Fig 5:**
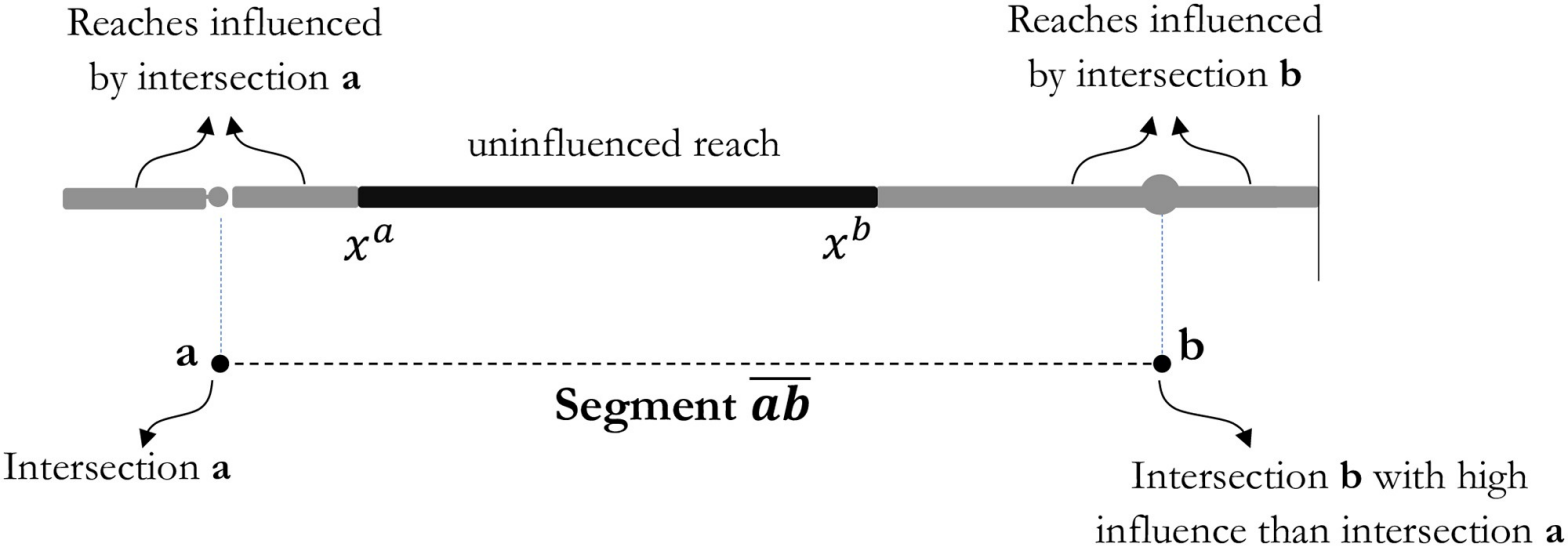
Different influential reaches of a segment.

Intersections are the end parts of the segments and are generally prone to different crash rates than other sections. A segment can thus be divided into three sections, the first and the last reach influenced by their closest intersections and the uninfluenced middle reach, as shown in Fig 5.

Assuming that the incident probability of the influential reach has a linear relationship with that of the uninfluential reach, the probability of the influential reach can be given using:

$$p_{\;}^{I}=\gamma p_{\;}^{U},$$ (8)

Where *p*^*I*^ and *p*^*U*^ are the *individual incident probabilities* of influential and uninfluential reach, respectively. *p*^*U*^ can be calculated using Eq (7). Values of *γ* can be acquired from past data or a field survey. Since the vehicle is in continuous motion during transportation, an incident at a particular location is only possible if the vehicle can reach that location. Therefore, the segment incident probability can be formulated using the’ system reliability’ concept. For example, If $q^{I^{1}},\;q_{U}$ and $q^{I^{2}}$ represent the first influential reach, middle uninfluential reach and second influential reach of the segment with independent individual incident probabilities $p_{\;}^{I^{1}},\;p^{U}$ and $p_{\;}^{I^{2}}$, respectively. The segment incident probability for a vehicle travelled successfully through the last reach $q^{I^{2}}$, is given as:

$$p_{q^{I^{2}}}=p_{\;}^{I^{1}}+\left(1-p_{\;}^{I^{1}}\right)p_{\;}^{U}+\left(1-p_{\;}^{I^{1}}\right)\left(1-p_{\;}^{U}\right)p_{\;}^{I^{2}},$$ (9)

Where ($1-p_{\;}^{I^{1}}$) and (1 − *p*^*U*^) are the probabilities that the incident did not happen on the first influential and middle uninfluential reach, respectively. Note that the incident probability of any point on the individual section of the segment is assumed to be the same as the individual incident probabilities of that section. One can also find the *locational incident probability* using the same concept, i.e., the probability of an incident at a specific location on the segment, by curtailing Eq (9). For example, if we intend to calculate locational incident probability at any location *x* in the middle uninfluential, the expression would be:

$$p_{x}=p_{\;}^{I^{1}}+\left(1-p_{\;}^{I^{1}}\right)p_{\;}^{U},$$ (10)

Eq (10) can be generalized to consider more influential reaches in the segment other than those near the intersection. For example, segments may have different individual incident probabilities at different locations or sections due to local adverse conditions. If a segment has *q* influenced reaches of varying length and different individual incident probabilities (or have a relationship as in Eq (8)), the segment incident probability can be given using:

$$p_{q}=p_{\;}^{1}+\left(1-p_{\;}^{1}\right)p_{\;}^{2}+\cdots +\left(1-p_{\;}^{1}\right)\left(1-p_{\;}^{2}\right)\ldots \left(1-p_{\;}^{q-1}\right)\;p_{\;}^{q},$$ (11)

We can also obtain the locational incident probability on the segment by curtailing Eq (11) as exampled in Eq (10). The use of locational incident probability in the proposed analysis is demonstrated in the coming sections. Refer to the approach in S2 File to allocate random accident locations based on individual incident probabilities.

*Hazard consequence*. We assume that the consequence of a HAZMAT event is a function of five factors, i.e., the consequence probability (*p*_*h*_), the magnitude of the potential hazard, the degree or amount of the assets exposed (*Q*), the assets’ vulnerability (*V*) against the released HAZMAT, and the total exposure time of the hazard (*H*). The magnitude of the hazard is a function of the intensity (*K*) of the released HAZMAT and its frequency (*U*). The quantification based on these factors can be given by:

$$\mathbb{C}=p_{h}\times K\times U\times Q\times V\times H,$$ (12)

Where *p*_*h*_ is the consequence probability (assumed equal to 1), as discussed in the previous section. As we are interested in a relative measure, we consider the consequence of the hazard’s one-time hitting; hence, the frequency can be taken as ‘1’ for a relative measure. Eq (12) can now be replaced by:

ℂ=K×Q×V×H, (13)

The assets’ vulnerability can be expressed as the ratio of the asset’s sensitivity toward the hazard to the safeguard measures against the hazard, which can take values equal to or greater than 0. The value of ‘0’ means the asset is fully protected and has no vulnerability or is not affected by the potential hazard, in which case the risk becomes zero. Values between 0 and 1 suggest that the asset is protected to some extent. The value of ‘1’ means the asset has normal sensitivity to the hazard and is not protected. The values greater than ‘1’ mean higher asset sensitivity towards the hazard and is considered under-protected. Note that by changing the value of the vulnerability ratio for assets, we can also measure the perceived risk. However, this relationship between vulnerability and perceived risk is linear. A power function can also be seen in the literature [53] by raising the risk consequence to a risk preference parameter.

The exposure time generally includes the time for post-incident efforts and treatments such as immediate evacuation and clearance. We can consider the response time from the nearest Emergency Response Service (ERS) Station for estimating the relative measure.

### Hazard area and types of analysis

The hazard area around an incident location can be a function of many factors, including the type of material (spread rate, intensity, chemical or radioactive nature), the topography of the surrounding region (terrain types, slopes, natural barriers), and meteorological conditions at the time of the incident (e.g., wind speed, and wind direction). Due to these numerous factors, an accurate determination of the consequence of the HAZMAT involved incident requires a lot of information. Therefore, simplification is necessary to be able to measure the risks justifiably. Literature review suggests that a circular area around the vehicle carrying HAZMAT is suitable for risk calculation [54]. It can be called a danger circle or a hazard circle. Yet, this simplification still presumes a uniform density of the assets falling within the danger circle, resulting in inaccuracies in risk estimation. It ignores the dynamic nature of the assets like population, the density of which may vary at different times of the day within the hazard circle. Nonetheless, keeping in view the complexities involved in estimating the precise consequences of a HAZMAT incident, this assumption is reasonable as also argued by [54].

**Fig 6:**
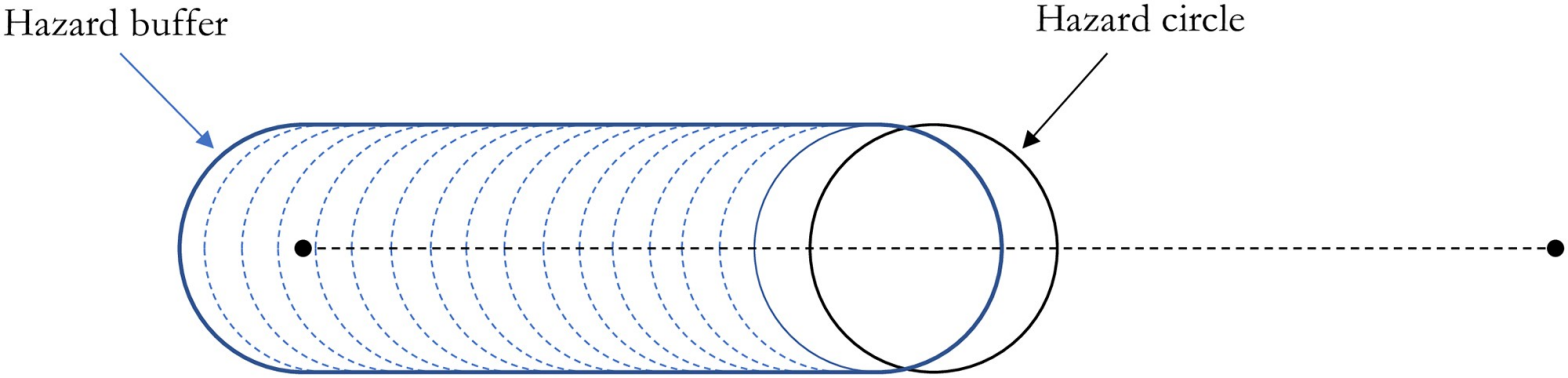
Hazard buffer and hazard circle along a segment.

Considering the same radius throughout the segment while the vehicle is traversing on the segment takes a form of a continuous buffer around the segment with a fixed distance from the segment called *hazard buffer* (Fig 6). Measuring the risk based on this constant hazard buffer indicates a rudimentary worst-case scenario analysis called *Deterministic Analysis* in the current article.

An assumption made in the hazard buffer approach is that all the assets located within the hazard buffer distance (*hazard buffer radius*) are exposed to the same hazard intensity, despite their locations within the hazard area (buffer). This assumption may not be realistic since the assets near the incident location will be exposed to a more severe hazard intensity than those far from the incident location. We tried to release this assumption at a broader scale by introducing the intensity-reducing factor for different asset agglomerations (population centers). The hazard’s intensity can vary depending upon the hazardous material being transported and can take many forms, from a simple distance measure to more complex functions (not within the current article’s scope). For simplicity and relative measurement over the network, we consider Hooke’s inverse-square law, which suggests that the intensity of a physical quantity decreases by the square of the distance away from the source of that physical quantity. It insinuates the following relationship:

$$\frac{K_{\text{location}\;a}}{K_{\text{location}\;b}}=\frac{{\left(\text{distance}\;\text{of}\;\text{location}\;\text{b}\;\text{from}\;\text{source}\right)}^{2}}{{\left(\text{distance}\;\text{of}\;\text{location}\;\text{a}\;\text{from}\;\text{source}\right)}^{2}},$$ (14)

If the reference location is taken one unit distance from the incident location, the intensity at the asset location can be given by:

Katassetlocation=1d2×Katoneunitdistance, (15)

Where 1d2 is the ratio, which is the intensity-reducing factor, and *d* is the Euclidian distance between the locations. Since the intensity level near the unit distance from the incident location is the reference and constant, we can safely eliminate it for relative measurement. Eq (13) can now be replaced by:

$$\mathbb{C}=\frac{1}{d^{2}}\times Q\times V\times H,$$ (16)

If *H* is in minutes, the consequence, hence the risk, takes the units of asset-minutes. In light of Eqs (11) and (16), the risk in Eq (3) can now be measured by:

$$\mathbb{R}=p_{q}\times \frac{1}{d^{2}}\times Q\times H\times V,$$ (17)

The asset that generally matters most in HAZMAT risk assessment is the human population in proximity to the incident location. The population can be quantified by multiplying the affected area by the population density of that area. The decision-makers may also want to protect the water bodies near a HAZMAT transport route. However, the risk estimation is comparatively complicated in case of water bodies, as the damage cannot be measured accurately after its first contact with the HAZMAT. For simplicity and relative measurement, the surface water’s projection area, which falls within the hazard area, is considered. In the deterministic analysis, since the buffer distance is constant, the risk to the population and water bodies using Eq (17) can be given by:

$${\mathbb{R}}^{Pop}=p_{s_{\left(e\right)}}\times \frac{1}{d_{s_{\left(e\right)}}^{2}}\times \left(A_{\Delta }^{B_{s_{\left(e\right)}}}{}_{\;}\times D_{\Delta }^{B_{s_{\left(e\right)}}}\right)\times H_{s_{\left(e\right)}}\times V_{\Delta }{}_{\;},$$ (18)

$${\mathbb{R}}^{w}=p_{s_{\left(e\right)}}\times \frac{1}{d_{s_{\left(e\right)}}^{2}}\times A_{\Upsilon }^{B_{s_{\left(e\right)}}}\times H_{s_{\left(e\right)}}\times V_{\Upsilon }$$ (19)

Where $p_{s_{\left(e\right)}}$ is the segment incident probability, $d_{s_{\left(e\right)}}^{2}$ is the nearest distance of the segment to the population center or water body, $A_{\Delta }^{B_{s_{\left(e\right)}}}$ is the area of the population center within the segment hazard buffer, ${A_{\Upsilon }^{B_{s_{\left(e\right)}}}}_{\;}$ is the area of the water body within the segment hazard buffer, $H_{s_{\left(e\right)}}$ is the time from segment centroid to the nearest ERS station, and *V*_Δ_ and *V*_*Υ*_ are the vulnerability ratio for the population center and the water body, respectively. Note that the risk is measured in persons-minute and area-minute for the population and for water bodies, respectively.

**Fig 7:**
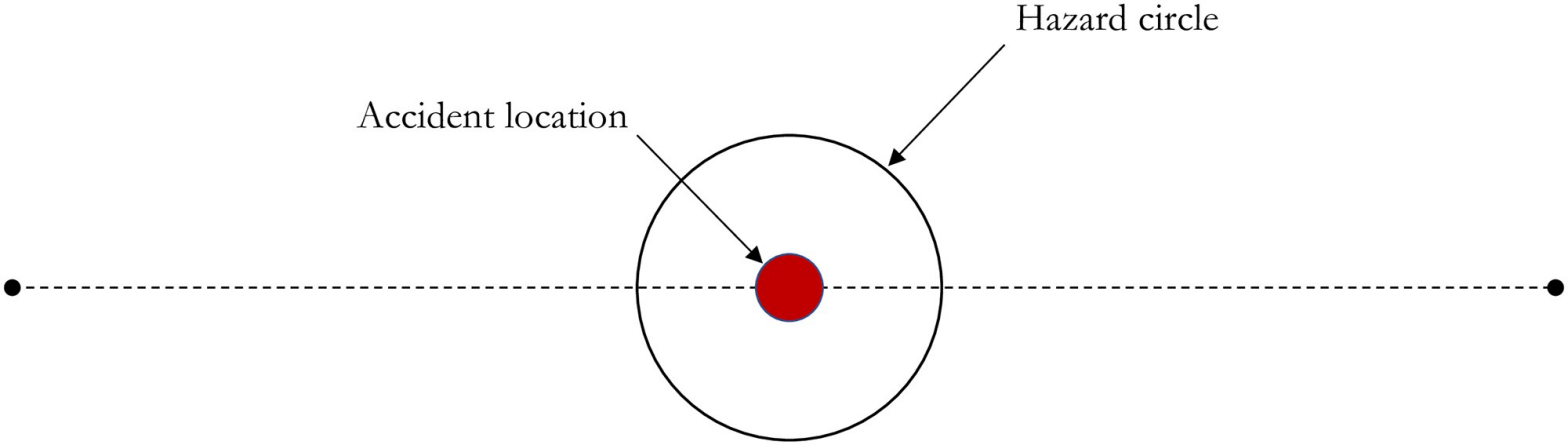
Hazard circle for an incident.

Risk analysis based on hazard buffer has a few more critical assumptions. For example, all the assets falling within the buffer are assumed to be prone to risk, which can overestimate the risk to a higher degree since in reality there could be a single incident on a particular segment with a randomly distributed location and maybe with a varied hazard area. Also, the number of assets within the incident’s actual hazard area may be drastically different from that of a buffer. Moreover, agencies take immediate preemptive measures to reduce the risk further after an incident. Another assumption is that all the assets located along a segment are exposed by the same likelihood, which may not be justified if the segment contains sections that are more prone to incidents than the rest of the segment reaches. Assets located near these more vulnerable sections are subject to risk with higher probability. To relax these assumptions, we propose the *Stochastic Analysis*, which aims to evaluate many scenarios to provide a range of outcomes based on uncertainties in input variables. For a specific scenario, the risk is measured at a random location on a segment, indicating that the incident can happen at any location on the segment in the real-world (Fig 7). For each scenario, the risk is measured only in a hazard circle at a particular location instead of a buffer around the whole segment. This reduces the chances of overestimation of the risk and is analogous to real situations. Also, as highlighted previously, the probability of the incident at a particular location on the segment may be different from that of the whole segment and may be calculated using Eqs (7)–(10). Therefore, in the stochastic analysis, the assets may be subject to different risks with different likelihoods. More details are presented about the stochastic analysis in the coming sections.

For stochastic analysis, risks based on random incident location can be determined by:

$${\mathbb{R}}^{Pop}=p_{s_{\left(x\right)}}\times \frac{1}{d_{s_{\left(x\right)}}^{2}}\times \left(A_{\Delta }^{B_{s_{\left(x\right)}}}{}_{\;}\times D_{\Delta }^{B_{s_{\left(x\right)}}}\right)\times H_{s_{\left(x\right)}}\times V_{\Delta },$$ (20)

$${\mathbb{R}}^{w}=p_{s_{\left(x\right)}}\times \frac{1}{d_{s_{\left(x\right)}}^{2}}\times A_{\Upsilon }^{B_{s_{\left(x\right)}}}{{}_{\;}}_{\;}\times H_{s_{\left(x\right)}}\times V_{\Upsilon },$$ (21)

Where $p_{s_{\left(x\right)}}$ is the locational incident probability at location *x*, $d_{s_{\left(x\right)}}^{2}$ is the nearest distance of the incident location to the population center or water body, ${A_{\Delta }^{B_{s_{\left(x\right)}}}}_{\;}$ is the area of the population center within the hazard circle at incident location *x*, ${A_{\Upsilon }^{B_{s_{\left(x\right)}}}}_{\;}$ is the area of the water body within the hazard circle at incident location *x*, and $H_{s_{\left(x\right)}}$ is the travelling time in minutes from the nearest ERS station to the incident location.

### Quantification of local-level relationship utilities

The local-level relationships form the basis for all higher-level relationships. Considering length as a segment attribute, the preference-based utility for a segment *s* using Eq (1) can be given as:

$$\delta_{\;s}^{\theta }={ϴ}_{s}\;\times l_{s},$$ (22)

Where, $\delta_{\;s}^{\theta }$ is the segment utility based on preference, *ϴ*_*s*_ is the mean preference score of the segment *s* and *l*_*s*_ is the length of the segment *s*. In light of Eqs (17)–(21), risk-based segment utilities for population centers and water bodies can be given as:

$$\delta_{\;s}^{\Delta }=p_{s_{\left(•\right)}}\times H_{s_{\left(•\right)}}\times \sum_{\Delta =1}^{m}\left(\frac{A_{\Delta }^{B_{s_{\left(•\right)}}}{}_{\;}\times D_{\Delta }^{B_{s_{\left(•\right)}}}}{d_{s_{\left(•\right)}}^{2}}\times V_{\Delta }\right),$$ (23)

$$\delta_{s}^{\Upsilon }=p_{s_{\left(•\right)}}\times H_{s_{\left(•\right)}}\times \sum_{\Upsilon =1}^{m}\left(\frac{A_{\Upsilon }^{B_{s_{\left(•\right)}}}{}_{\;}}{d_{s_{\left(•\right)}}^{2}}\times V_{\Upsilon }\right),$$ (24)

Where *s*_(•)_ denotes variable value either for the whole segment (deterministic) or for an incident location (stochastic) depending upon the type of analysis under consideration, *m* and k are the total number of population centers or water bodies inside the hazard area, respectively.

**Fig 8:**
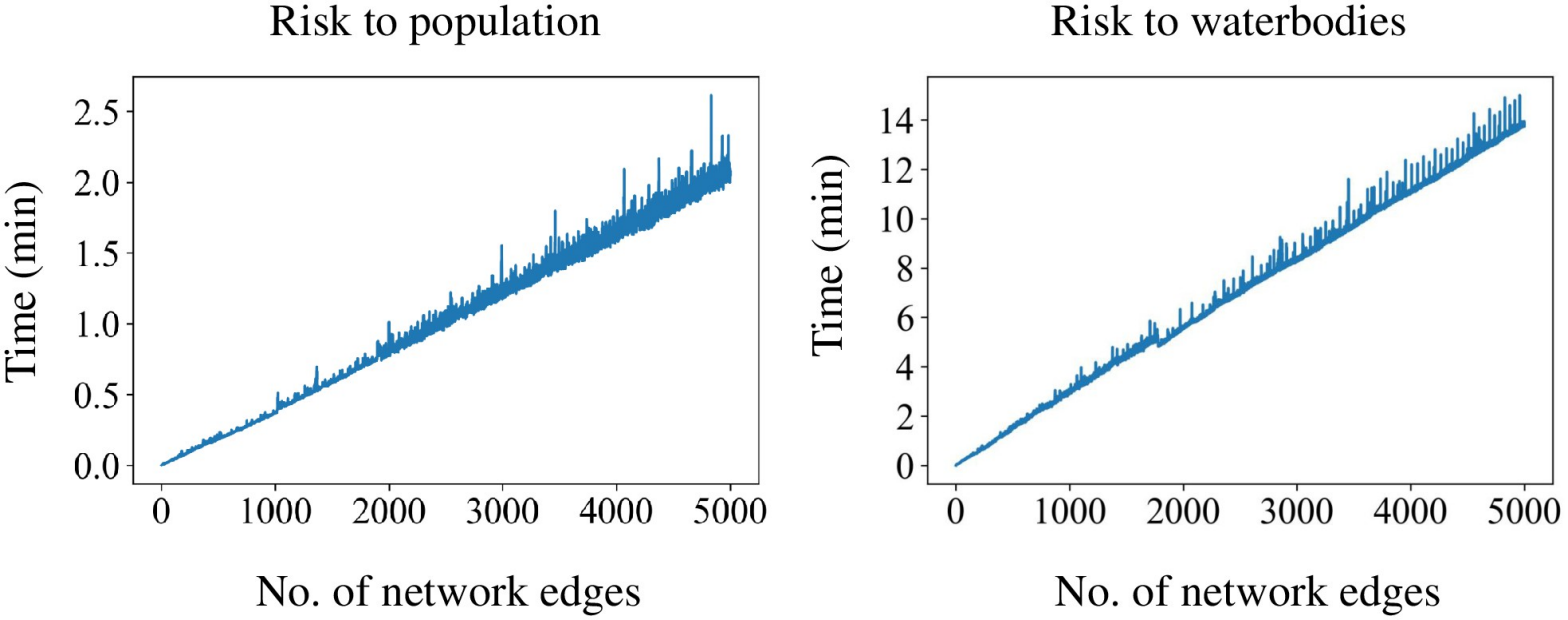
Computational complexity to calculate local-level utility.

The linear complexity ***O***(|S|) of these utility functions (Eqs (22)–(24)) is given in Fig 8, considering the hazard radius of 11Km, with 711 population polygons and 11,593 lake polygons and river lines. Where |*S*| is the number of segments in the network. With |*C*| number of criteria, the complexity becomes ***O***(|C||S|). The complexity calculated is based on steps in S5 File. The actual complexity can change with varying number of assets and the hazard radius.

### Development of route-level relationship utilities

The route-level relationship stems from solving the routing problem in the network for each POI-zone pair. If the problem is to find the routes between designated supply and demand sites, it can be solved by applying algorithms that can generate simple paths. If the problem is to find the shortest routes, the shortest path algorithm can be used. Depending upon the routing problem and objectives, route-level relationships can be developed. For a single route problem, the route-level relationship utility can be given by:

$$\zeta_{{Ф}_{oz}^{r}}^{c}{{}_{\;}}_{\;}={\left(\sum_{\begin{array}{c} \;\\ s=1,\\ \left(s\in U\right)\\ \left(U≙{Ф}_{oz}^{r}\right) \end{array}}^{n}\delta_{\;s}^{c}\right)}_{{Ф}_{oz}^{r}},$$ (25)

Where *n* is the number of segments in the route ${Ф}_{oz}^{r},\;c$ is the criteria for which the relationship is sorted, and *r* is the criteria based on which the routing problem is solved. Note that *r* can be the same as *c* if the routes are developed based on the same criteria for which the local and route-level relationships are developed. This implies that the routes developed for one criterion can be considered to develop route-level relationships for another criterion. The illustration below would help.

For the current analysis, a “preferred” multi-modal transportation network can be established, considering segment preference scores as the only criterion (which can be retrieved through a survey study, as discussed earlier). Since the objective is to find the most preferred route with associated minimum risk (maximum risk-based utilities), the first task is to find the route which provides the most preferred segments. In other words, it is intended to find the routes from each POI to each zone that contains the segments with maximum segment-level relationship utilities concerning the decision-makers’ preference, i.e. max ($\delta_{\;s}^{\theta }$). If the preference for a segment category is represented numerically by the lowest number on the scale, the maximum utility would be given by min ($\delta_{\;s}^{\theta }$). The route-level utility based on the preferences of segment categories can be determined by finding a route for each POI-zone pair, i.e., *preferred route*, and can be calculated as:

$$\zeta_{\varphi_{oz}^{\theta }}^{\theta }{{}_{\;}}_{\;}={\left(\sum_{\begin{array}{c} \;\\ s=1,\\ \left(s\in U\right)\\ \left(U≙\varphi_{oz}^{\theta }\right) \end{array}}^{n}\left({ϴ}_{s}\;\times l_{s}\right)\right)}_{\varphi_{oz}^{\theta }}\;\;,$$ (26)

Where $\varphi_{oz}^{\theta }$ is the most preferred route (*best route*) between a POI *o* and zone *z* calculated by the shortest path algorithm. Similarly, the risks posed by each segment can be calculated on these preferred routes using risk functions, and route-level utilities based on risk to the population and water bodies can be calculated (considering the same routes) as follows, respectively:

$$\zeta_{\varphi_{oz}^{\theta }}^{\Delta }={\left(\sum_{\begin{array}{c} \;\\ s=1,\\ \left(s\in U\right)\\ \left(U≙\varphi_{oz}^{\theta }\right) \end{array}}^{n}\left(p_{s_{\left(•\right)}}\times H_{s_{\left(•\right)}}\times \sum_{\Delta =1}^{m}\left(\frac{A_{\Delta }^{B_{s_{\left(•\right)}}}{}_{\;}\times D_{\Delta }^{B_{s_{\left(•\right)}}}}{d_{s_{\left(•\right)}}^{2}}\times V_{\Delta }\right)\right)\right)}_{\varphi_{oz}^{\theta }},$$ (27)

$$\zeta_{\varphi_{oz}^{\theta }}^{\Upsilon }={\left(\sum_{\begin{array}{c} \;\\ s=1,\\ \left(s\in U\right)\\ \left(U≙\varphi_{oz}^{\theta }\right) \end{array}}^{n}\left(p_{s_{\left(•\right)}}\times H_{s_{\left(•\right)}}\times \sum_{\Upsilon =1}^{m}\left(\frac{A_{\Upsilon }^{B_{s_{\left(•\right)}}}{}_{\;}\times D_{\Upsilon }^{B_{s_{\left(•\right)}}}}{d_{s_{\left(•\right)}}^{2}}\times V_{\Upsilon }\right)\right)\right)}_{\varphi_{oz}^{\theta }},$$ (28)

Where *n* is the number of segments in the preferred route from a POI *o* to a zone *z*.

### Development of zone-level relationship utilities

In developing zone-level relationships, more than one route or a combination of different routes from POIs may be considered depending upon the decision-makers’ need or choice. However, considering the number of evaluation criteria, the same number of zone-level relationships are needed for each zone, i.e., one for each criterion. Zone-level utilities for a zone can be quantified as follows:

$$\Omega_{z}^{c}=\Sigma_{o=1}^{O}\zeta_{\Phi_{oz}^{r}}^{c},$$ (29)

Where $\Omega_{z}^{c\;}$ is the zone-level relationship utility for zone *z* for criteria *c*, and *O* is the set of POIs. Note that zone-level utility accounts for all route-level utilities from all POIs for a particular zone (collective relationship). The values of these zone-level utilities, assigned as attributes to each zone, form the basis for siting optimization. In the current analysis, the zone-level utilities for route preference, population risk and risk to water bodies can be formulated as follows, respectively:

$$\Omega_{z}^{\theta }=\Sigma_{o=1}^{O}{\left(\sum_{\begin{array}{c} s=1,\\ \left(s\in U\right)\\ \left(U≙\varphi_{oz}^{\theta }\right) \end{array}}^{n}\left({ϴ}_{s}\times l_{s}\right)\right)}_{\varphi_{oz}^{\theta }},$$ (30)

$$\Omega_{z}^{\Delta }=\Sigma_{o=1}^{O}{\left(\sum_{\begin{array}{c} s=1,\\ \left(s\in U\right)\\ \left(U≙\varphi_{oz}^{\theta }\right) \end{array}}^{n}\left(p_{s_{\left(•\right)}}\times H_{s_{\left(•\right)}}\times \sum_{\Delta =1}^{m}\left(\frac{A_{\Delta }^{B_{s_{\left(•\right)}}}\times D_{\Delta }^{B_{s_{\left(•\right)}}}}{d_{s_{\left(•\right)}}^{2}}\times V_{\Delta }\right)\right)\right)}_{\varphi_{oz}^{\theta }},$$ (31)

$$\Omega_{z}^{\Upsilon }=\Sigma_{o=1}^{O}{\left(\sum_{\begin{array}{c} s=1,\\ \left(s\in U\right)\\ \left(U≙\varphi_{oz}^{\theta }\right) \end{array}}^{n}\left(p_{s_{\left(•\right)}}\times H_{s_{\left(•\right)}}\times \sum_{\Upsilon =1}^{m}\left(\frac{A_{\Upsilon }^{B_{s_{\left(•\right)}}}\times D_{\Upsilon }^{B_{s_{\left(•\right)}}}}{d_{s_{\left(•\right)}}^{2}}\times V_{\Upsilon }\right)\right)\right)}_{\varphi_{oz}^{\theta }},$$ (32)

### Siting optimization

The ranking of the zones based on multiple attributes is a multi-objective optimization problem. As a matter of fact, any optimization criteria for optimality can be adopted for the proposed stochastic method. We demonstrated the methodology using the Pareto-optimal concept because of its simplicity and the fact that it relies on the unbiased trade-off among different criteria giving equal preference to each criterion. The decision-maker does not have to worry about the weights for each criterion, which is a big advantage. Please refer to S4 File for the concept of Pareto-optimality, the algorithm to find Pareto-optimal solutions, and its complexity.

The problem can also be formulated in a standard multi-objective optimization framework as an unconstrained multi-objective optimization problem, as follows:

$${\text{min}}_{z}f_{\theta }\left(z\right)={\text{min}}_{z}\Omega_{z}^{\theta }={\text{min}}_{z}\sum_{o=1}^{O}{\left(\sum_{\begin{array}{c} \;\\ s=1,\\ \left(s\in U\right)\\ \left(U≙\varphi_{oz}^{\theta }\right) \end{array}}^{n}\left(\theta_{s}\times l_{s}\right)\right)}_{\varphi_{oz}^{\theta }},$$ (33)

$${\text{min}}_{z}f_{\Delta }\left(z\right)\;={\text{min}}_{z}\Omega_{z}^{\Delta }={\text{min}}_{z}\sum_{o=1}^{O}{\left(\sum_{\begin{array}{c} \;\\ s=1,\\ \left(s\in U\right)\\ \left(U≙\varphi_{oz}^{\theta }\right) \end{array}}^{n}\left(p_{s_{\left(•\right)}}\times H_{s_{\left(•\right)}}\times \sum_{\Delta =1}^{m}{\left(\frac{A_{\Delta }^{B_{s_{\left(•\right)}}}{}_{\;}\times D_{\Delta }^{B_{s_{\left(•\right)}}}}{d_{s_{\left(•\right)}}^{2}}\times V_{\Delta }\right)}_{\;}\right)\right)}_{\varphi_{oz}^{\theta }},$$ (34)

$${\text{min}}_{z}f_{\Upsilon }\left(z\right)={\text{min}}_{z}\Omega_{z}^{\Upsilon }={\text{min}}_{z}\sum_{o=1}^{O}{\left(\sum_{\begin{array}{c} \;\\ s=1,\\ \left(s\in U\right)\\ \left(U≙\varphi_{oz}^{\theta }\right) \end{array}}^{n}\left(p_{s_{\left(•\right)}}\times H_{s_{\left(•\right)}}\times \sum_{\Upsilon =1}^{m}{\left(\frac{A_{\Upsilon }^{B_{s_{\left(•\right)}}}{}_{\;}\times D_{\Upsilon }^{B_{s_{\left(•\right)}}}}{d_{s_{\left(•\right)}}^{2}}\times V_{\Upsilon }\right)}_{\;}\right)\right)}_{\varphi_{oz}^{\theta }},$$ (35)

A basic descent method can solve this unconstrained optimization problem. However, the problem can also be defined with constraints on route-level and zone-level utilities as follows:

$$\zeta_{*}^{\theta }\geq \zeta_{\varphi_{oz}^{\theta }}^{\theta },$$ (36)

$$\zeta_{*}^{\Delta }\geq \zeta_{\varphi_{oz}^{\theta }}^{\Delta },$$ (37)

$$\zeta_{*}^{\Upsilon }\geq \zeta_{\varphi_{oz}^{\theta }}^{\Upsilon },$$ (38)

$$\Omega_{*}^{\theta }\geq \Omega_{z}^{\theta },$$ (39)

$$\Omega_{*}^{\Delta }\geq \Omega_{z}^{\Delta },$$ (40)

$$\Omega_{*}^{\Upsilon }\geq \Omega_{z}^{\Upsilon },$$ (41)

Where $\zeta_{*}^{\theta \;},\zeta_{*}^{\Delta \;}$ and $\;\zeta_{*}^{\Upsilon }$ are the threshold route-level utilities and $\Omega_{*}^{\theta \;},\Omega_{*}^{\Delta \;}\&\;\Omega_{*}^{\Upsilon \;}$ are threshold zone-level utilities set by the decision-maker for preference, population risk and water risk criteria, respectively. Eqs (36)–(38) set the upper limit for a route-level utility in case the routes are not considered feasible beyond this utility limit. Similarly, Eqs (39)–(41) set limits for the zone-level utilities in case the total utility for a zone is not acceptable beyond this limit. Eqs (26)–(28) and (30)–(32) defines the values of $\zeta_{\varphi_{oz}^{\theta }}^{\theta \;},\;\zeta_{\varphi_{oz}^{\theta }}^{\Delta \;}\;$ and $\;\zeta_{\varphi_{oz}^{\theta }}^{\Upsilon \;}$, and $\Omega_{z}^{\theta \;},\;\Omega_{z}^{\Delta \;}$ and $\;\Omega_{z}^{\Upsilon \;}$, respectively.

#### Conflicting objectives and global optimality

In terms of utility, the intention is to increase the utility in each objective criterion. However, the meaning of utility is not the same for each criterion. For instance, the preference-based utility increases with the shortest paths if the paths contain highly preferred segments. On the other hand, the utility based on population and water risk increases when those shortest paths pose the least risk to population and water areas. Qualitatively speaking, the preference should be maximized, but risks should be minimized, i.e., the objectives are qualitatively conflicting. Quantitatively, all objectives are to be minimized because, in the proposed methodology example, a higher preference towards network segment categories is represented by a lower number on the survey scale. Thus, it is intended to minimize the preference function given in Eq (33). Please note that the optimal Pareto solutions are based on unbiased trade-offs between the objective functions. Finding these trade-off solutions is an ideal strategy for multi-objective optimization rather than a preference-based strategy that combines all criteria into a single objective problem [55]. If the objectives are not in conflict, the multi-objective optimization problem produces only one Pareto-optimal solution since there would not be any trade-off. However, multiple solutions are guaranteed when the objectives are in conflict. It is also important to mention that the solutions obtained from this strategy are globally optimal. Consider the following definition by [55]:

*Definition*. *The non-dominated set of the entire feasible search space S is the globally Pareto-optimal set*.

The Pareto-optimal set is a globally Pareto-optimal set, implying that the solutions obtained as Pareto-optimal are not dominated by any other solution in the feasible search space and are globally optimal solutions.

### Deterministic and stochastic analyses

In a deterministic analysis, i) all the equations for local-level utility are restricted to a fixed hazard buffer distance. Assets within a predefined hazard zone are considered, and the portions of the assets inside the hazard zone are then considered for the quantification of the local-level utility (Eqs (23) and (24)), and ii) the incident probability of the segment is used in the relationship functions. Network loading is done with new values at a segment level by assigning the relationship functions to the segments, which forms the basis for relationship utility. These utilities are used instead of initial segment lengths to solve the routing problem. While calculating the route-level utility based on preference scores, there are two outcomes; the routes which form the basis for risk-based route-level utilities and the route-level utility based on decision-makers’ preferences. This preference-based route-level utility and risk-based route-level utilities contribute to the zone-level utility and is assigned as a zone attribute for the optimization process.

The stochastic analysis takes random incident locations and suggestively arbitrary hazard circle size on each segment. In this regard, the locational incident probability is required, unlike in deterministic analysis, where the segment incident probability is considered. This change gives stochastic analysis advantages over deterministic analysis as the risk is calculated in concurrence with a real situation. Only the assets within the incident location’s hazard area are subject to the risk. One can set a range of hazard circle radii to encounter the release and effect probability for a HAZMAT type. For instance, different hazard areas corresponding to different hazard circle radii may imply different amounts of effective releases. Since finding the release and effect probability is challenging, as discussed previously, this technique can help. Taking a random buffer for the same type of HAZMAT implies that a random amount of HAZMAT is released each time an incident is modelled. A closed range of hazard circles can be set depending on the type of material for this purpose. Finding the ‘release and effect probability’ along the total reach of the route is highly uncertain and rather unpredictable. Therefore, any assumption made in this regard should be backed up with a priori study, i.e., based on a priori information available to the decision-maker on the expected HAZMAT to be transported and other factors such as road condition, any proper distribution (that makes more sense statistically or strategically) can be used. An example of such a priori effort can be seen in the emergency response guidebook 2020 by the US Department of Transport and Transport Canada [56] prepared for use by first responders during the initial phase of a transportation incident involving hazardous materials. It can be noted in this guidebook that a circular area (buffer circle) is used to represent isolation distances/zones, and material vapour plumes are used for protective action zones for different hazardous materials, keeping in view wind direction, among other factors. Indeed, this is a complicated task and is out of the scope of the current study.

**Fig 9:**
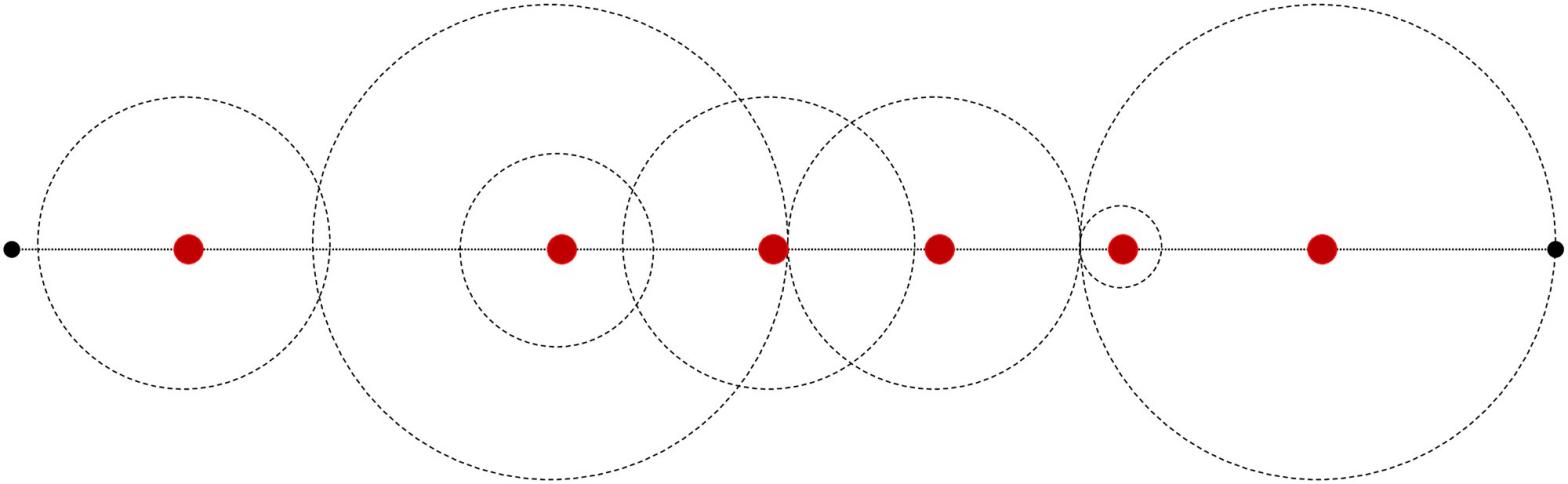
Example of hazard area based on random location and random hazard circle.

**Fig 10:**
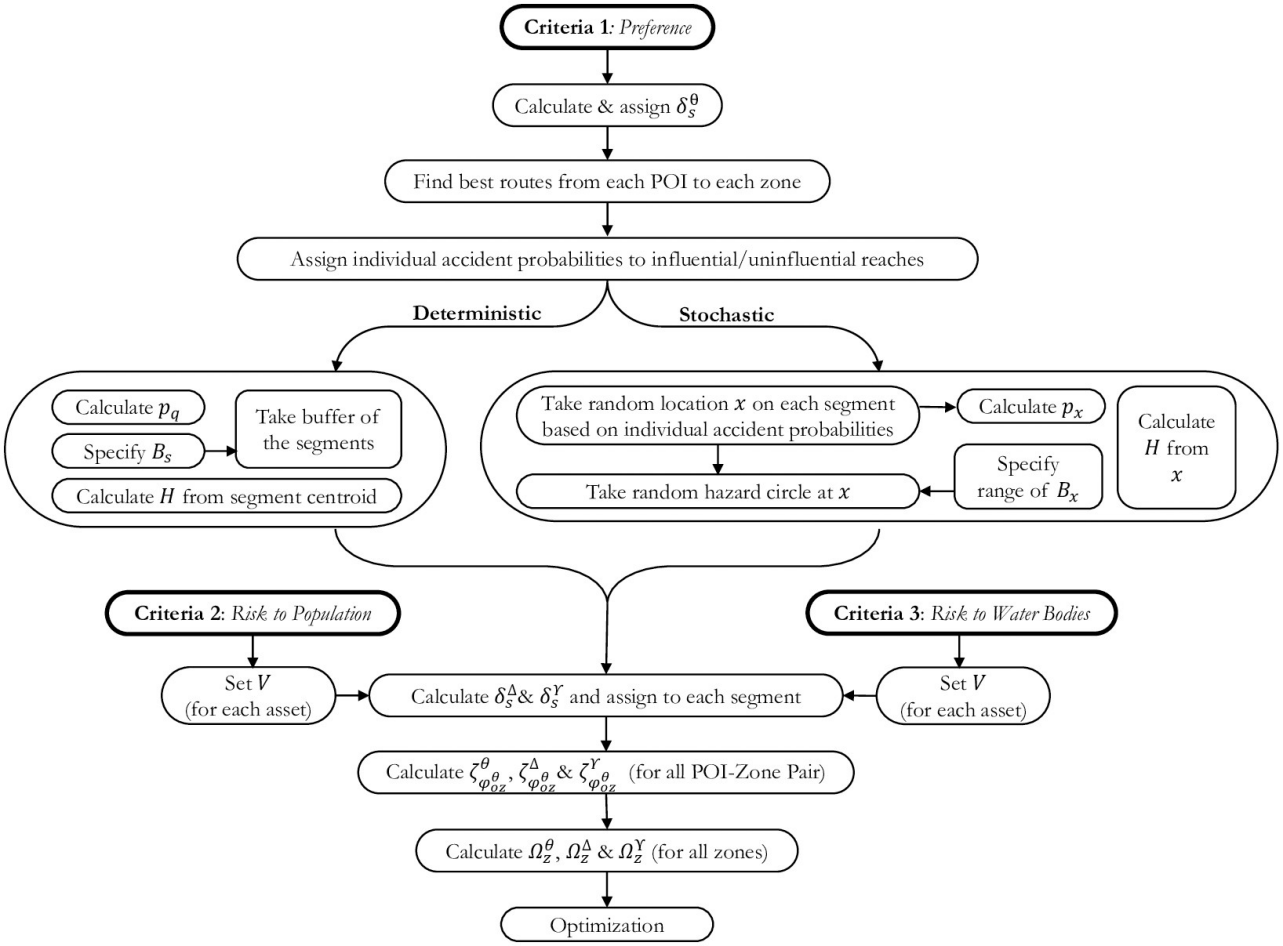
Current methodology highlighting the difference in risk measurement approaches.

In practical cases, the type of HAZMAT to be transported would most probably be known, and the hazard circle radius can be carefully selected, which can be of different sizes at different locations, keeping in view the safety factor to be applied in connection to the type of accident to be expected or the packaging quality of the HAZMAT, among other criteria such as wind direction and speed or the road conditions at different sections of the road. Due to such limitations, a fixed buffer has often been used in the literature to estimate the potential risk or the consequences. Fig 9 shows a pictorial view of random hazard circles and hypothetical incident locations for a typical network segment. Varying hazard circle radius can also be used to handle different HAZMAT types. However, it can also be fixed for a typical HAZMAT, which refers to a uniform constant release and effect probability. Fig 10 describes the methodology for the current analysis highlighting the difference in deterministic and stochastic risk measurement to develop the local-level relationships and subsequent higher-level relationships.

#### Stochastic risk estimation

A HAZMAT facility, if located at the wrong site, may lead to long-term consequences beyond the means envisaged at the time of planning since regular shipments will form part of the system, and actual scenarios are highly uncertain. The stochastic analysis proposed in this research aims to quantify the risks realistically. The improved risk functions (Eqs (20) and (21)) allow incorporating hazard exposure time. The exposure time increases if the emergency aid does not reach the accident’s location in time; therefore, a location-specific treatment is required to avoid underestimation or overestimation of the risk. The stochastic analysis measures the risk strictly at the accident location to avoid this, which is a clear improvement in calculating the risks. Also, please note that the vulnerability factor *V* introduced in the revised risk functions controls sensitivity towards the risk, which can be used as a data-driven safety factor in a controlled manner, avoiding any blind overestimation. On the other hand, overestimating the risk by deterministic analysis can be misleading in selecting a site. For example, an optimal site calculated based on deterministic analysis may not be optimal for many of the actual risk scenarios or a series of accidents that happened in due course of time, as the risk to surrounding features depends on the accident locations and the associated hazard area. Therefore, whenever the location of the accident changes, the risk scenario changes and hence the optimality of the site. Consequently, it cannot be concluded with surety that the optimal sites calculated based on the deterministic analysis will always be optimal. Therefore, we adopt the stochastic approach to rank the sites based on their optimal behaviour. By simulating accidents and observing the long-term optimality of the site, we can be comparatively more certain about the site choice. In this sense, stochastic analysis is a reasonable approach that avoids under or overestimating the risk [57]. discusses the concerns on the overestimation of risk by using a semicircular buffer for risk estimation and the possibility of selecting the wrong path compared to a relatively improved buffer area for more accurate risk measurement.

#### Advantages of the stochastic approach

The proposed stochastic approach is adopted to find a diverse set of optimized solutions by repeating the optimization process. The actual strength of the proposed stochastic method comes from three aspects: the comparatively more accurate estimation of the risk; the possibility of a greater number of diversified optimal zones; and the ranking of optimal zones. The risk calculation is close to realistic when we simulate accident locations instead of buffering around the network segment. The number of optimal zones increases based on the introduction of more risk scenarios, as we will discuss in the coming sections. Apart from this, stochastic treatment unfolds the ranking of the sites based on how frequently they appear as optimal sites every time a new risk scenario is introduced as per the accident probabilities.

### Zone ranking

In the case of deterministic analysis, the sites that are found optimal are considered superior, yet all with the same standing (non-dominated sites) as compared to non-optimal zones (dominated sites). Since the sites are not further ranked, we conclude that the sites are classified into two categories, e.g., ‘suitable’ or optimal and ‘unsuitable’ or non-optimal, implying that all suitable sites are ranked equally. However, in stochastic analysis, finding whether a site is optimal is just one instance that corresponds to a single risk scenario considered on the preferred route. The risk scenario corresponds to random accidents on each route segment with different locations and possibly different hazard circles, resulting in a specific population and water risk utilities. By repeating the analysis considering different risk scenarios, we seek to know how often a site found optimal (i.e., in how many risk scenarios the site stands optimal). Since each risk scenario results from a stochastic process, it may lead to a different set of optimal zones each time. Therefore, we test the stability of the zones for their optimality (i.e., the probability of a zone being optimal in response to multiple risk scenarios). By doing so, we get rankings of the “optimal zones”. An optimal zone is ranked better than other optimal zones if its probability of optimality is higher, i.e., it remains optimal in more yet diverse risk scenarios.

The proposed ranking concept has practical significance. For example, if a top-ranked optimal site could not be used due to any practical reasons (e.g., geological or environmental factors), the decision-maker will have the flexibility to select the next optimal site with a better probability of optimality than other remaining sites. This flexibility is beneficial when the decision-maker needs to consider additional selection criteria imposed by other disciplines such as geological, land use, or other site selection factors and investigate the trade-off among different sites based on additional external criteria. The process can be thought of as using a piece of higher-level information to find a solution from obtained trade-off (optimal) solutions, which is concurrent with an ideal strategy to solve a multi-objective optimization problem as indicated by [58].

#### Ranking based on the collective relationship

Based on the optimization results, the zones will be ranked for collective relationship. Non-dominated solutions straightforwardly give the best zones in the case of deterministic analysis. However, in the case of stochastic analysis, considering the repetitive analyses for different scenarios of the incident location and hazard areas, the performance measure to rank a zone is the number of times a zone is identified as a Pareto Optimal solution in the total number of random trials. The zones that were found to be Pareto Optimal more frequently are considered superior.

**Fig 11:**
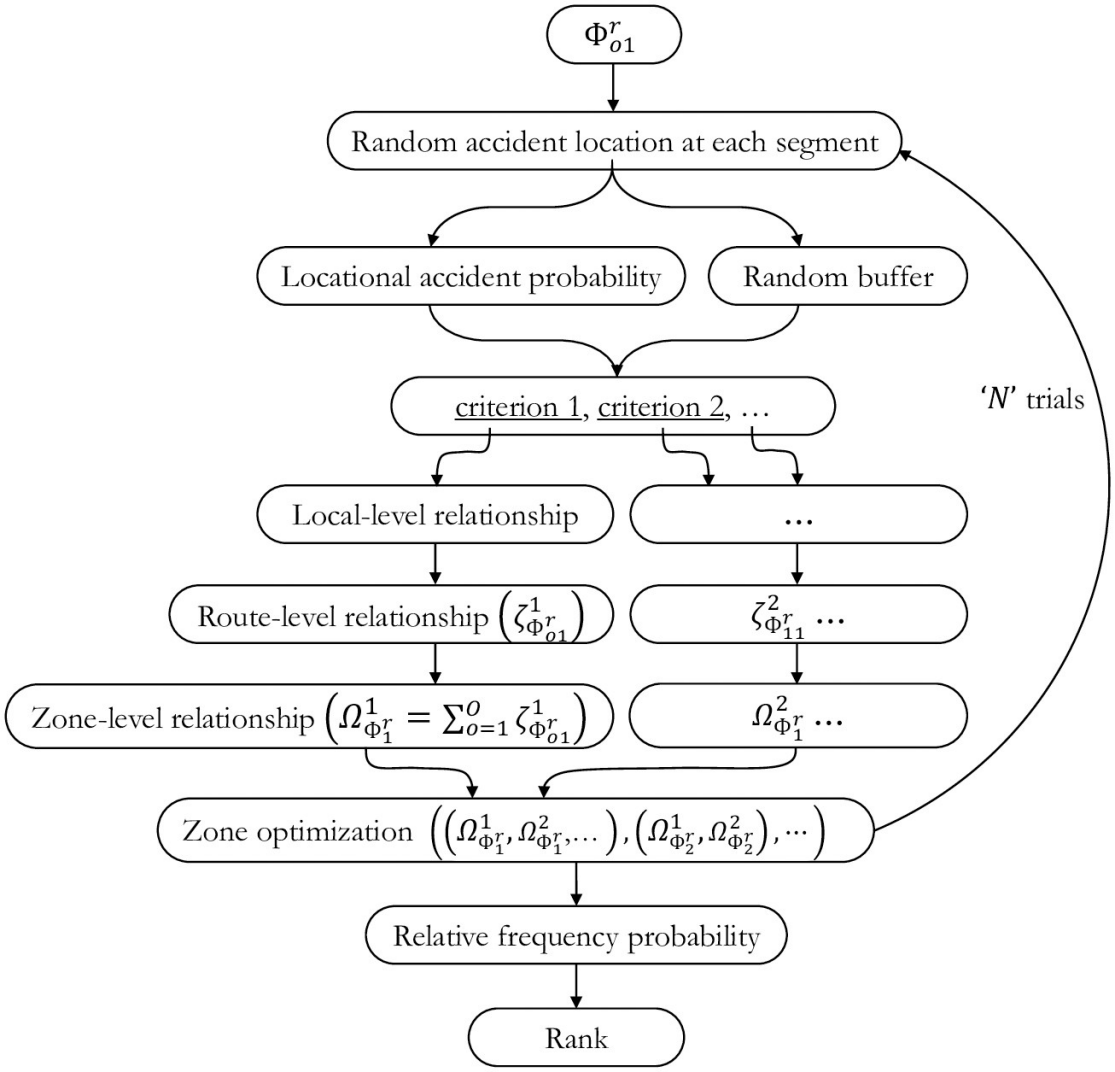
Ranking of zones based on stochastic analysis.

Each stochastic trial assigns a binary label to each zone: either ‘0’ indicating that a zone does not fall on a *Pareto-frontier* (a failure), or ‘1’ that the zone does fall on a Pareto-frontier (a success). Suppose we replicate the experiment several times. In that case, the proportion of the successes, i.e., the number of times a zone falls on a Pareto Frontier, can be calculated considering all experiments (stochastic trials). In probability theory, the law of large numbers suggests that the probability of the occurrence of an event can be approximated by the proportion of the number of occurrences if the experiment is repeated over many times. Using this axiom, we can calculate the probability of a zone falling on a Pareto Frontier for a random process by repeating it multiple times. The zones can be ranked based on this probability. Fig 11 presents the flow for ranking the zones.

Mathematically, this probability of optimality can be expressed as:

$${ƥ}_{z}^{\;}=\frac{1}{N}\overset{N}{\underset{i=1}{\sum }}S_{z}^{i},\;\left\{\begin{array}{ll} S_{z}^{i}=1, & \text{if}\;z\;\text{is}\;\text{on}\;\text{Pareto}-\text{frontier}\\ S_{z}^{i}=0, & \text{if}\;z\;\text{is}\;\text{not}\;\text{on}\;\text{Pareto}-\text{frontier} \end{array}\right.$$ (42)

Where **ƥ**_***z***_ is the probability of optimality of zone *z*, *N* is the number of iterations, $S_{z}^{i\;}$ is the status of the candidate zone *z* after the optimization process in the *i*^th^ iteration. The status of the candidate zone ($S_{z}^{\;}$) can be represented by:

$$S_{z}=⟴\left(\Omega_{z}^{c_{1}},\Omega_{z}^{c_{2}},\;\Omega_{z}^{c_{3}},\ldots \Omega_{z}^{c_{n}}\right),$$ (43)

Where ⟴ is the optimization operator used in the optimization process and *c*_1_, *c*_2_, *c*_3_, …, *c*_*n*_ ∈ *C* are evaluation criteria under consideration. For the current analysis, $S_{z}^{\;}=⟴(\Omega_{z}^{\theta \;},\Omega_{z}^{\Delta \;},\;\Omega_{z}^{\Upsilon \;})$.

#### Ranking based on the one-to-one relationship

Similar to the method we adopted for ranking on a collective relationship basis, we can also rank zones based on one-to-one relationships considering all evaluation criteria. A zone will become a Pareto Optimal candidate by assigning route-level utility from each criterion to that zone, and zones are compared on an individual POI basis. However, stochastic trials give the probabilities for each zone against each POI in that case. Ranking zones based on these individual probabilities can be an advantage in two cases, first, if a certain POI or more POIs are given preference, and second, if the zones are to be ranked based on the optimal behaviour for a maximum number of POIs. The probability of optimality for the one-to-one relationship can be calculated using Eq (35). The status of the candidate zone for optimality for one-to-one relationship is represented by:

$$S_{oz}^{\;}=⟴\left(\zeta_{\Phi_{oz}^{r}}^{c_{1}},\zeta_{\Phi_{oz}^{r}}^{c_{2}},\;\zeta_{\Phi_{oz}^{r}}^{c_{3}},\ldots \zeta_{\Phi_{oz}^{r}}^{c_{n}}\right)$$ (44)

For the current analysis, $S_{oz}^{\;}=⟴(\zeta_{\varphi_{oz}^{\theta }}^{\theta \;},\zeta_{\varphi_{oz}^{\theta }}^{\Delta \;},\;\zeta_{\varphi_{oz}^{\theta }}^{\Upsilon \;})$.

#### Stochasticity in preference

It is pertinent to mention that the optimal sites in the current analysis are determined based on the preferred network that is formed by definite preferred routes from each POI to each zone. The results depend on these routes as we fixed the routes based on preference scores and measured the risk on these routes only. Suppose it is intended to consider the variation in the preferences of the decision-makers. In that case, the procedure could be applied to the new set of preference scores to get a different set of optimal sites with new associated routes. It is also possible to consider stochasticity in preference values while determining the preferred network, in that case, (the routes will not be fixed as each iteration will give a new set of routes for a POI-Zone pair.

## Application and results

### Preparation of network and spatial models

The proposed analysis method was applied to the Canadian province of Saskatchewan to find optimal sites for a potential nuclear facility (e.g., fuel fabrication facility). Most of Canada’s uranium reserves are located in northern Saskatchewan, which forms the world’s largest high-grade deposits.

The network of the study area is a multimodal network comprised of railways and roads. The National Road Network (NRN) [59] and National Railway Network (NRWN) [60] spatial data were used. For demonstration purposes, it was assumed that both sub-networks (NRN and NRWN) are connected only at two locations to form a multimodal network, one in the city of Regina at Global Transportation Hub and the other on in the city of Saskatoon at the Canadian National Railway freight terminal. These locations were designated as multimodal freight terminals (only transfer points for freight in the whole network). It was assumed that NRWN and multimodal freight terminals are available for HAZMAT transportation, irrespective of their ownership, operational or legal status. NRN dataset contains essential data for the road segments such as length, functional classification, speed, number of lanes and geographical coordinates. The NRWN dataset contains information on railway segments such as operational tracks and categories in terms of freight and passenger. The network was converted to an undirected graph, assuming each segment has two-way movement.

**Fig 12:**
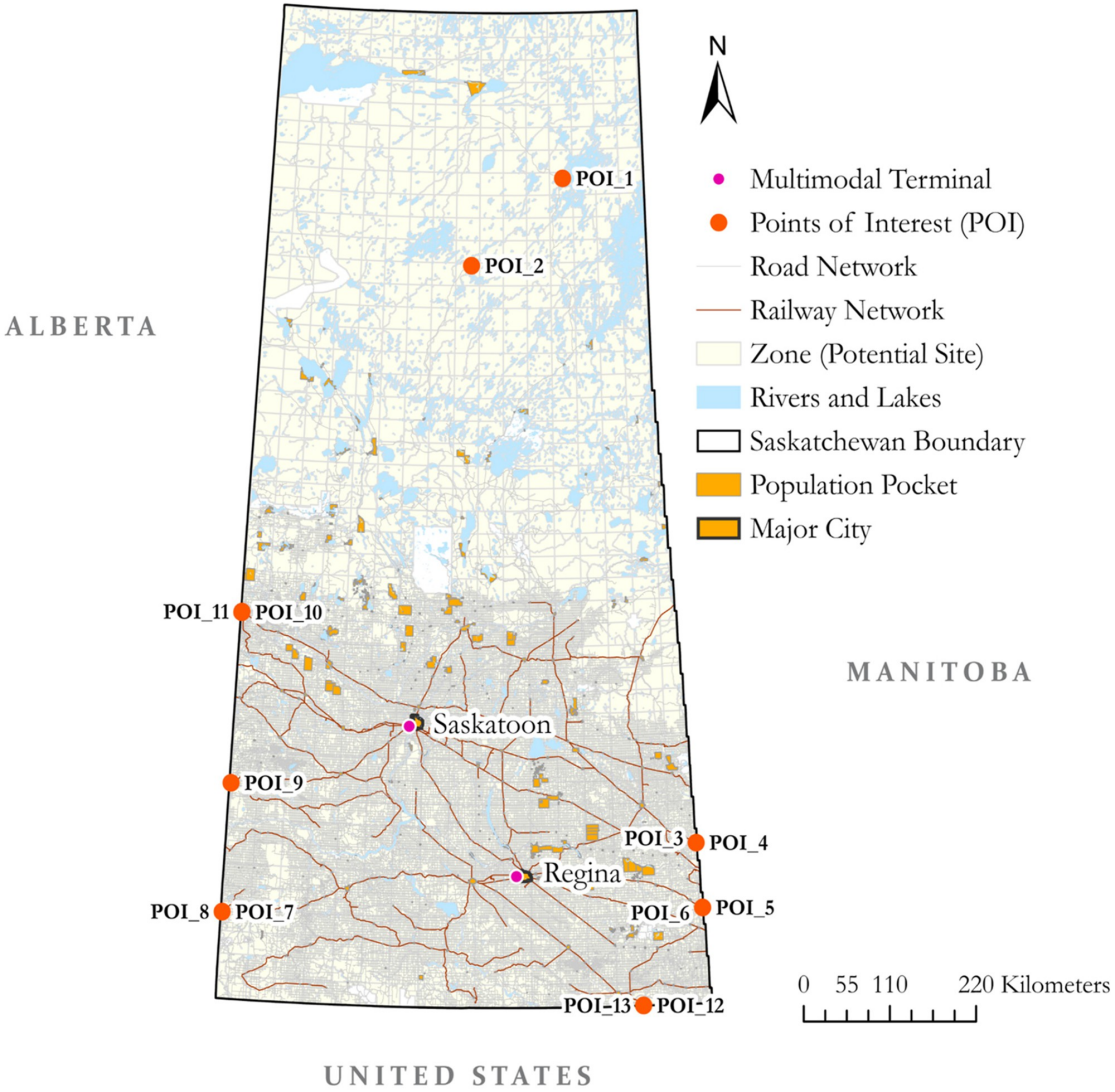
The Canadian province of Saskatchewan (study area). The map is generated using the data retrieved from [59–62] and contains information licensed under the Open Government Licence-Canada or Statistics Canada Open Licence.

The grid produced by the National Topographic Manning System (NTS) was adopted which divides the whole study area into rectangular zones. Each zone was considered as a potential candidate site for the nuclear facility. Spatial data regarding population centers and water bodies were used from the Statistics Canada website [61]. All these geographical data were transformed to NAD83 / UTM zone 13N projection. A total of 823 zones were identified after clipping out all sensitive areas such as lakes, rivers, population centers, and reserve parks. The zones were represented by their centroids and connected to the network via hypothetical local access roads with a speed of 40 km/hr. For demonstration purposes, 13 locations were designated in the network as potential POIs. These POIs are selected as the connection to the adjacent provinces on the main provincial highways and railway tracks for interprovincial transportation. POIs 1 and 2 were located in the northern region of the province as potential query sites for nuclear materials. POIs 3 through 11 were considered along interprovincial highways and railways at Manitoba (Highways 1, Highway 16, and adjacent railway lines along both highways) and Alberta borders (Highway 1, Highway 7, Highway 16, and adjacent railroads along Highway 1 and Highway 16). Finally, POIs 12 and 13 were considered at the United States border along Highway 39 (border crossing point) and the adjacent railway line, respectively, for a potential transportation of the nuclear related material from outside Canada. Fig 12 shows the map for the study area along with all mentioned geographical features.

### Crash rates and incident probabilities

Incident rates were calculated using Eq (5), considering available AADT data for segments. The network was divided into rural and urban categories by clipping the road network using census metropolitan area (CMA) boundaries of the province [62] to apply SPFs (and relevant coefficients) for urban and rural areas [52]. Due to the limitations of the data, only AADT for highways was available. Synthetic AADT was used in light of Highway Functional Classification Concepts, Criteria and Procedures [63] for missing segment data. Similarly, train-miles (in millions) were used for railway segments attached to railway crossings. Train movement data available for railway crossing data was obtained from the Government of Canada-Open Data portal [64] and converted to billion train-miles. SPF for railway segments and related coefficients (*α* = 97.53, *β* = −0.05 and *θ* = 3.45) were used as per Eq (6) as suggested by [65, 66] based on 15 years of train collision data of the United States from 2001 to 2015. The year index of 2020 was used. Refer to the S3 File for the details regarding limitations of the data, data processing and fixtures applied to complete the data for the application.

Incident probabilities were calculated based on crash rates assuming Poisson’s distribution. For the sake of demonstration, the value of *γ* equal to ‘1’ was used (Eq (8)), i.e., uniform incident probability throughout the segment as calculated using Poisson’s distribution. In this case, the segment incident probability and locational incident probabilities are the same for the segments.

### Hazard area limits and exposure time

A value of 11 km was used for buffer distance as well as for the hazard circle radius range. This value was adopted (for radioactive materials) in light of the 2020 Emergency Response Guidebook [56] for demonstration purposes. As explained in Section 3.10, a closed range of values can be used for a typical HAZMAT type under consideration to handle release and effect probabilities. Hazard exposure time was calculated using speed and length data available for each road segment in NRN. For railway segments, the exposure time was calculated using speeds of 64 km/hr for railway segments passing through the population centers and 80 km/hr elsewhere [66]. Exposure time for the deterministic analysis was calculated from the centroid of the segment and from the point of the random incident for the stochastic analysis.

### Decision-makers’ preferences on route planning

A survey was used to incorporate the decision-makers’ preference in route planning, conducted in August 2018 from different experts of different relevant government departments of the province of Saskatchewan. The experts were asked about their preferences on rail and different road categories to transport nuclear material safely. The scale used for the survey represented the score of ‘1’ to reflect the highest preference and ‘10’ to reflect the lowest preference. Initially, there were 14 segment categories, which were reduced to 9 using the Chi-Square test for the current analysis. Categories of lower hierarchy that did not have a significant difference in the preference scores were merged. Table 5 represents the mean preference scores used for each category. The preference-based local-level relationships were calculated using Eq (22) by applying these mean preference scores and assigned to each network segment. As a next step, the best routes (most preferred) were calculated from each POI to each zone. The best route is represented by the minimum cost route (i.e., preference score considered as cost) in the current analysis since the scale used for preference scores presents high preference as a lower value. The accumulated local-level relationship utilities of these individual routes represent the route-level relationship between each zone and the POI and were calculated using Eq (26), which provide $\zeta_{\varphi_{oz}^{\theta }}^{\theta }$ values for each POI-Zone pair. Since the analysis is based on the minimum cost route (i.e., most preferred), only one route per POI-Zone pair was considered. These routes are labelled as *preferred routes*, and the risks to the population and water bodies were estimated on these routes only.

**Table 5:** Segment categories and decision-makers’ preferences.

Road categories	Preference score (mean)
**Railroad**	2.00
**Freeway**	2.88
**Highway (paved)**	3.05
**Highway (unpaved)**	5.00
**Arterial (paved)**	5.25
**Arterial (unpaved)**	6.48
**Collector (paved)**	6.88
**Collector (unpaved)**	7.60
**Local**	9.10

## Results and discussion

We performed three different analyses to demonstrate the proposed methodology and see the difference in the location and number of optimal zones; 1) Deterministic with the weighted-sum approach, 2) Deterministic with Pareto-optimization, and 3) Stochastic with Pareto-optimization. The preference-based routes developed earlier are common for each case analysis.

### Deterministic with the weighted-sum optimization approach

In the first case, the risk on the preferred routes’ segments is calculated deterministically, and the weighted-sum method is used for siting optimization. In the weighted-sum approach, all the objectives are converted to either a minimization problem or a maximization problem and each criterion is given normalized weightage as per their importance in light of the decision-makers’ point of view. Mathematically, these weights are multiplied to the respective values calculated for the criteria under consideration, and the values of weighted criteria are added up to form a single-objective optimization problem. This method results in one optimized solution. For demonstration purposes, fixed hazard buffer distances (HBD) of 11, 5, 1 and 0.5 km were considered to calculate the risk using Eqs (18) and (19). Table 6 presents the weights for route preference, population risk and risk to water bodies used for demonstration, respectively.

**Table 6:** Weights for each criterion for the weighted-sum approach.

Weights (Route preference, Population risk, Water risk)	Preferences
**(1:1:8)**	Highest preference to water risk
**(1:8:1)**	Highest preference to population risk
**(8:1:1)**	Highest preference to route preference
**(0.33,0.34,0.33)**	Almost equal preference to all criteria

**Fig 13:**
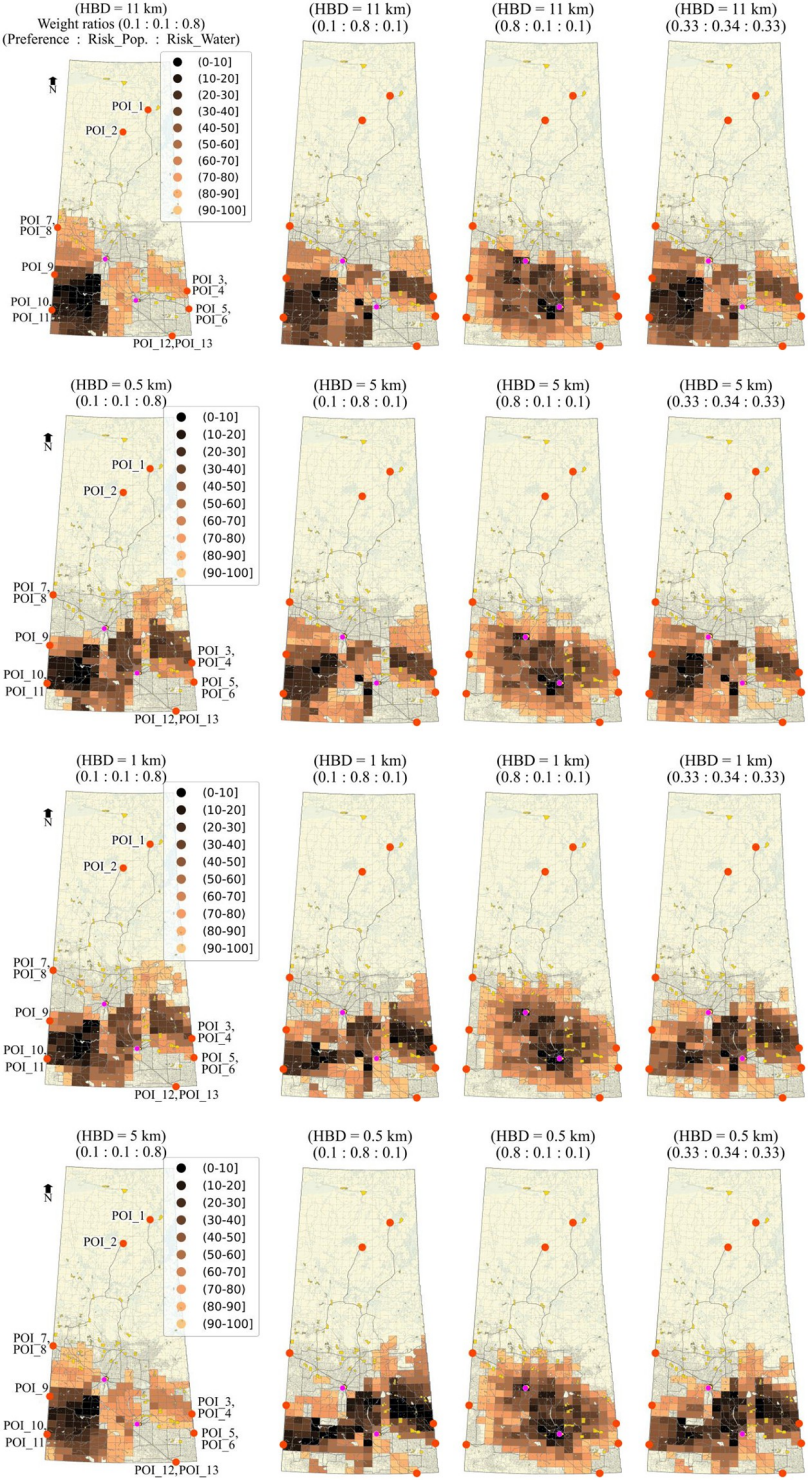
Heat map showing the top 25% suitable zones for case 1 analysis.

Note that Eqs (18) and (19) were applied to the preferred routes. The weights mentioned in Table 6 were applied to the zone-level relationship utilities calculated using Eqs (26)–(28) and (30)–(32). Since the method assigns varying values to the zones, the results can be shown in the form of a heat map. For clarity and effectiveness, only the top 25% of the zones are displayed. Fig 13 presents the results for the deterministic approach based on the weighted-sum approach. The legend mentions the normalized values for the zones. The lowest values represent the most suitable zone in light of the current analysis. The heat map highlights that the suitability of the zones changes based on the weights given to each criterion; also, the suitable zones change with buffer distance. It can be noted that although the ranking of zones based on the weighted-sum method is straightforward, determining the weight factors is not trivial. In addition, the analysis does not consider the stochasticity involved in risk analysis.

### Deterministic with pareto-optimization

**Fig 14:**
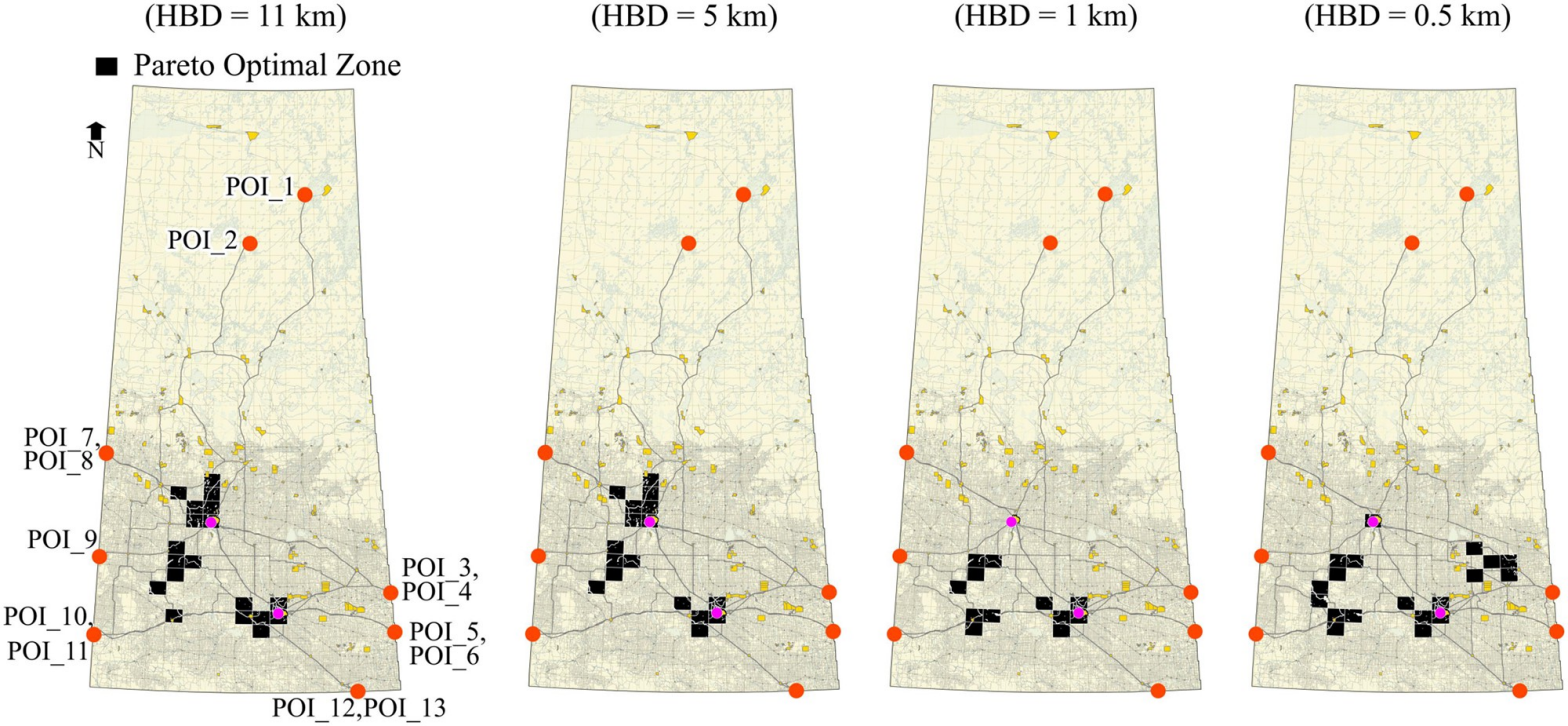
Heat map showing optimal zones for case 2 analysis.

The second case refers to the deterministic risk analysis to rank the zones based on the multi-objective Pareto optimization method. Risks using Eqs (18) and (19) were estimated considering HBDs of 11, 5, 1 and 0.5 km around segments of the preferred routes, separately. Eqs (26)–(28) and (30)–(32) were used to calculate relationships utilities up to the zone level. Based on three criteria zone-level utilities, i.e., route preference, population risk, and risk to water bodies, the Pareto optimal zones were determined using Eqs (33)–(35) and Eqs (45)–(46) in Supporting Information S4, as the sorting principle. Fig 14 shows the Pareto optimal zones for different buffer distances.

The heat maps show 19, 17, 12 and 18 optimal zones for 11, 5, 1 and 0.5 km buffer distances, respectively. It can be noticed that different buffer distances result in a different set of optimal zones since the number of population centers and water bodies within each hazard buffer is different. These optimal zones represent suitable (i.e., Pareto-optimal) zones considering all three criteria.

**Fig 15:**
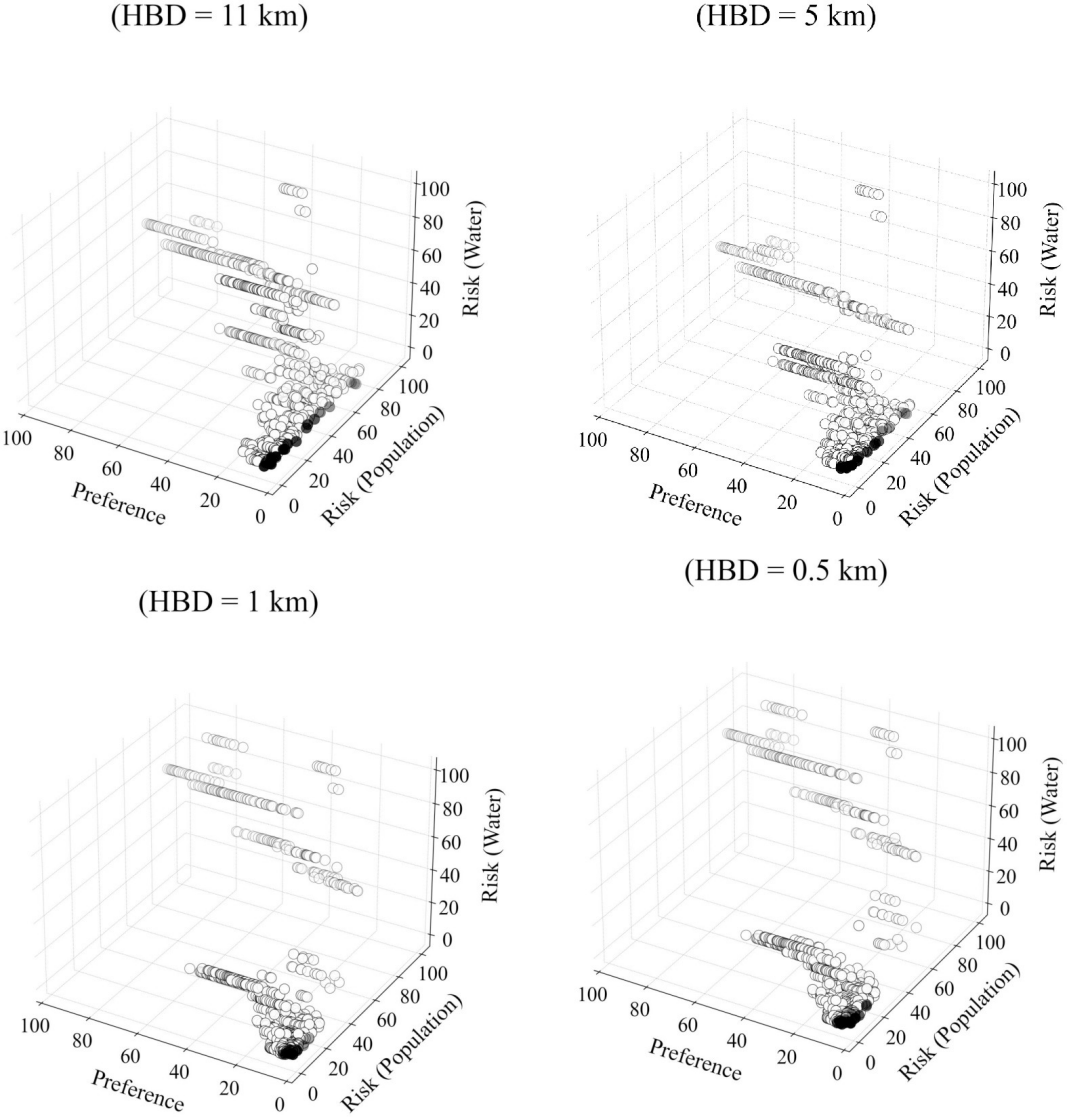
Pareto chart for case 2 analysis.

It can be seen from Fig 14 that the objectives are in conflict since the solutions are more than one. Fig 15 shows a 3D Pareto chart for the zones. It can be seen from the chart that the optimal zones get closer to each other when the buffer radius is reduced. This is mainly due to the increasingly consistent population risk within the reduced buffers. Since water bodies are mostly spread away from most of the preferred routes, they are not inflicting much difference.

### Stochastic with pareto-optimization

**Fig 16:**
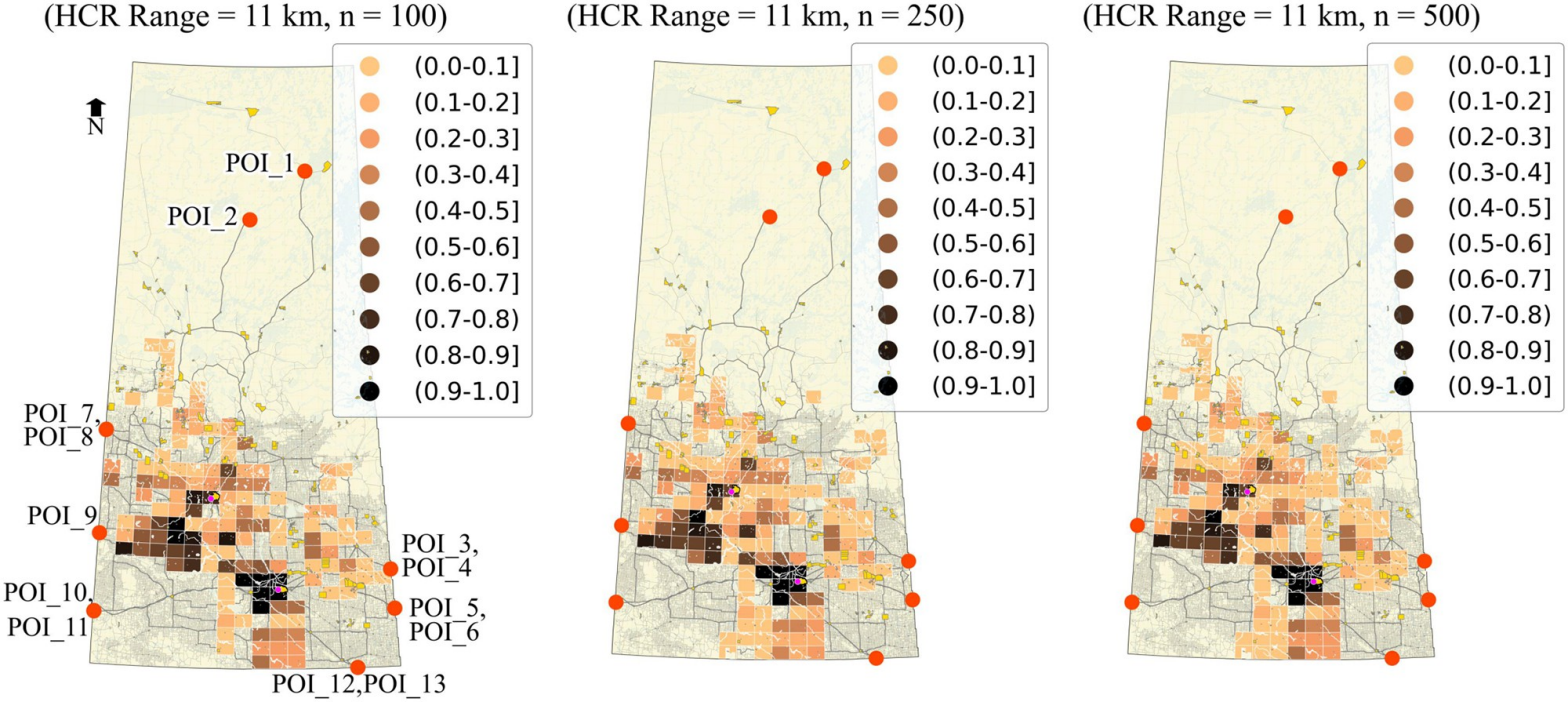
Heat map showing the probability of optimality of zones for case 3 analysis.

**Fig 17:**
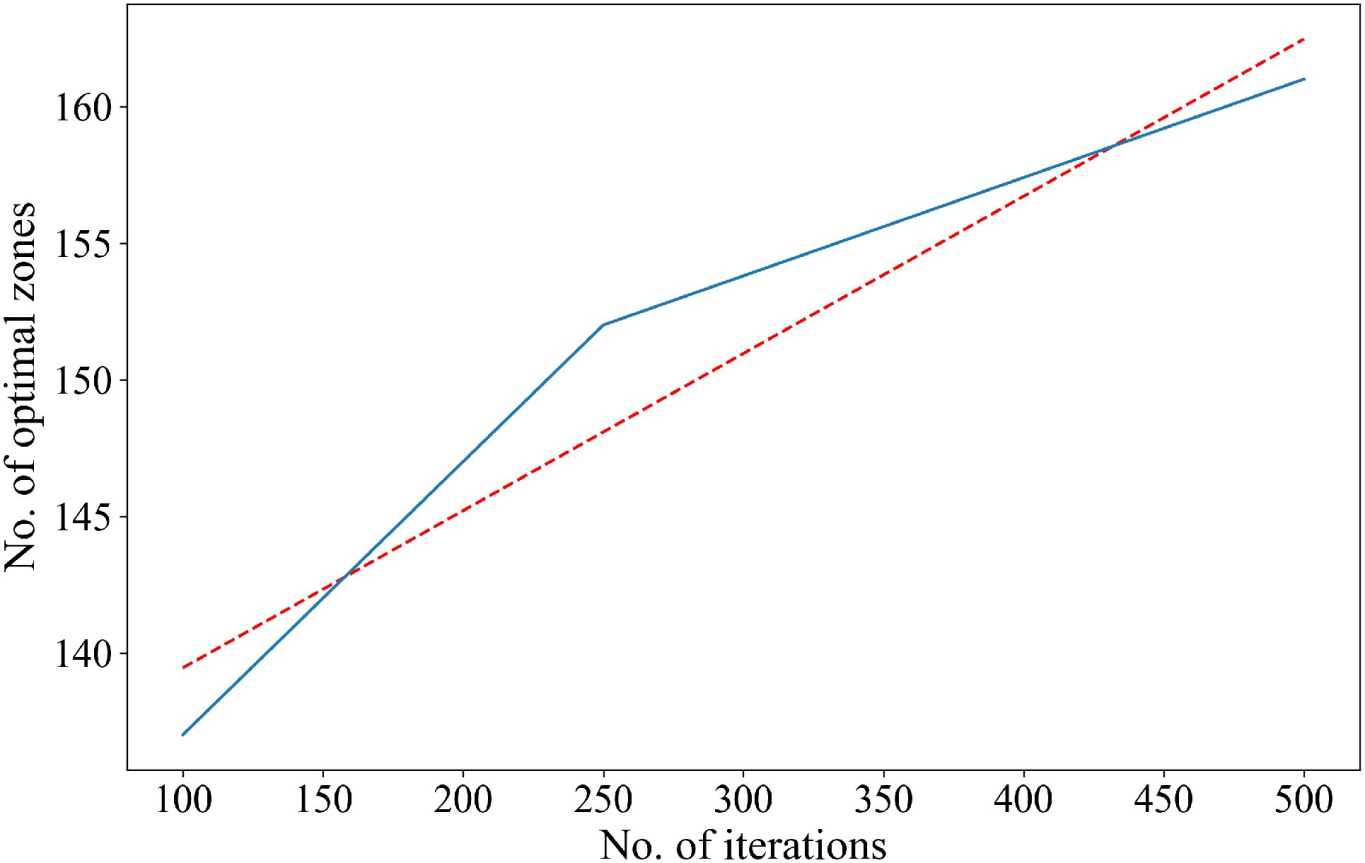
Plot showing number of optimal zones against no. of simulations.

The third case refers to the optimization of the siting based on Pareto optimization; however, the measurement of the risk and ranking of the zones is based on a stochastic process. Random incidents were considered on each segment of the preferred routes, and the incident locations were assigned according to segment incident probabilities. Locational incident probabilities were assumed to be the same as segment incident probabilities due to data limitations. Refer to the steps in S1 Fig as described in S2 File to determine accident location based on individual accident probabilities. Two analyses were performed, i.e., for a range of hazard circle radius (HCR), and for a fixed HCR. The risks of the segments were measured using Eqs (23) and (24). The route-level relationship utilities were calculated using Eqs (26)–(28), and the zone-level relationship utilities were calculated using Eqs (30)–(32). Siting optimization was performed using Eqs (33)–(35) and Eqs (45)-(46) Supporting Information S4, and the probability of optimality was calculated using Eqs (42) and (43). Three sets of iterations were made to see the effect of stochasticity in risk scenarios. Fig 16 shows the Pareto optimal zones with a uniformly distributed random HCR ranging from ‘0’ to ‘11’ km for 100, 250 and 500 iterations. The colour scheme shows the probability of the optimality for each optimal zone. Fig 17 shows the relation between the number of iterations and the number of zones that appear on the Pareto Frontier. The number of optimal zones increases by an increase in the number of iterations; however, the increase rate is higher for the first 250 iterations, indicating stability of the results as the number of iterations increase.

**Fig 18:**
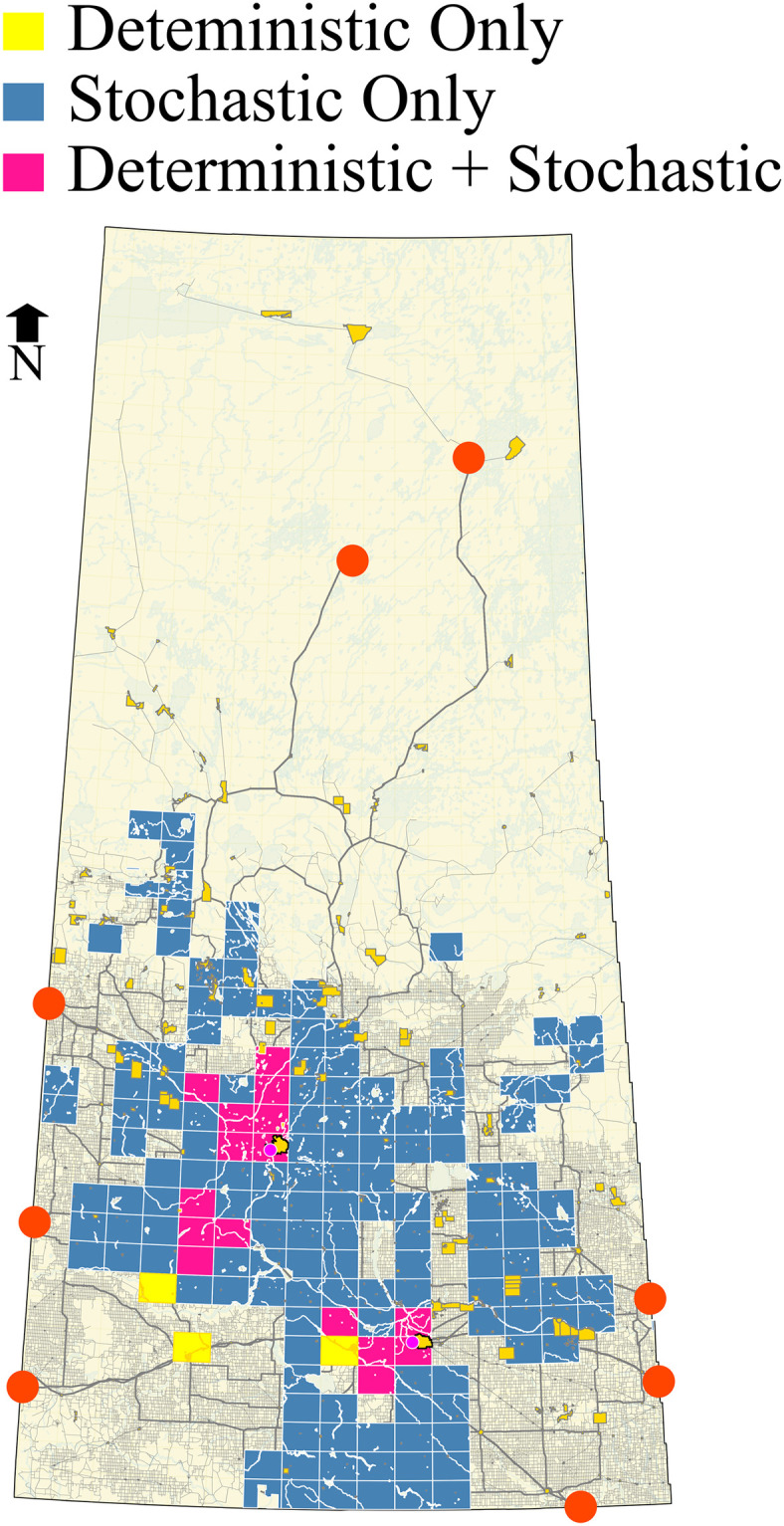
Heat map comparison between cases 2 and case 3 analyses. The map is generated using the data retrieved from [59–62] and contains information licensed under the Open Government Licence-Canada or Statistics Canada Open Licence.

It can be seen from the figure that the number of optimal zones has drastically increased as compared to case 2 in Fig 14 due to considerations for stochasticity in risk analysis. It is important to mention here that each optimal zone is the result of a particular risk scenario. Zones that appear on Pareto Frontier more frequently are more desirable. Therefore, the probabilities of optimality were calculated by repeating the iterations (risk scenarios). It can be noted that the number of optimal zones has increased slightly with the increase in the number of trials. Also, the clustering of the optimal zones as observed in case 2 analysis is dispersed; however, some optimal zones with a higher probability of optimality still hold the same location. The results indicate that the stochastic analysis can provide reasonable outcomes even without increasing the number of trials (i.e. computationally efficient). Fig 18. compares the deterministic and stochastic (considering 500 iterations) results for the 11km HBD and HCR range, respectively.

**Fig 19:**
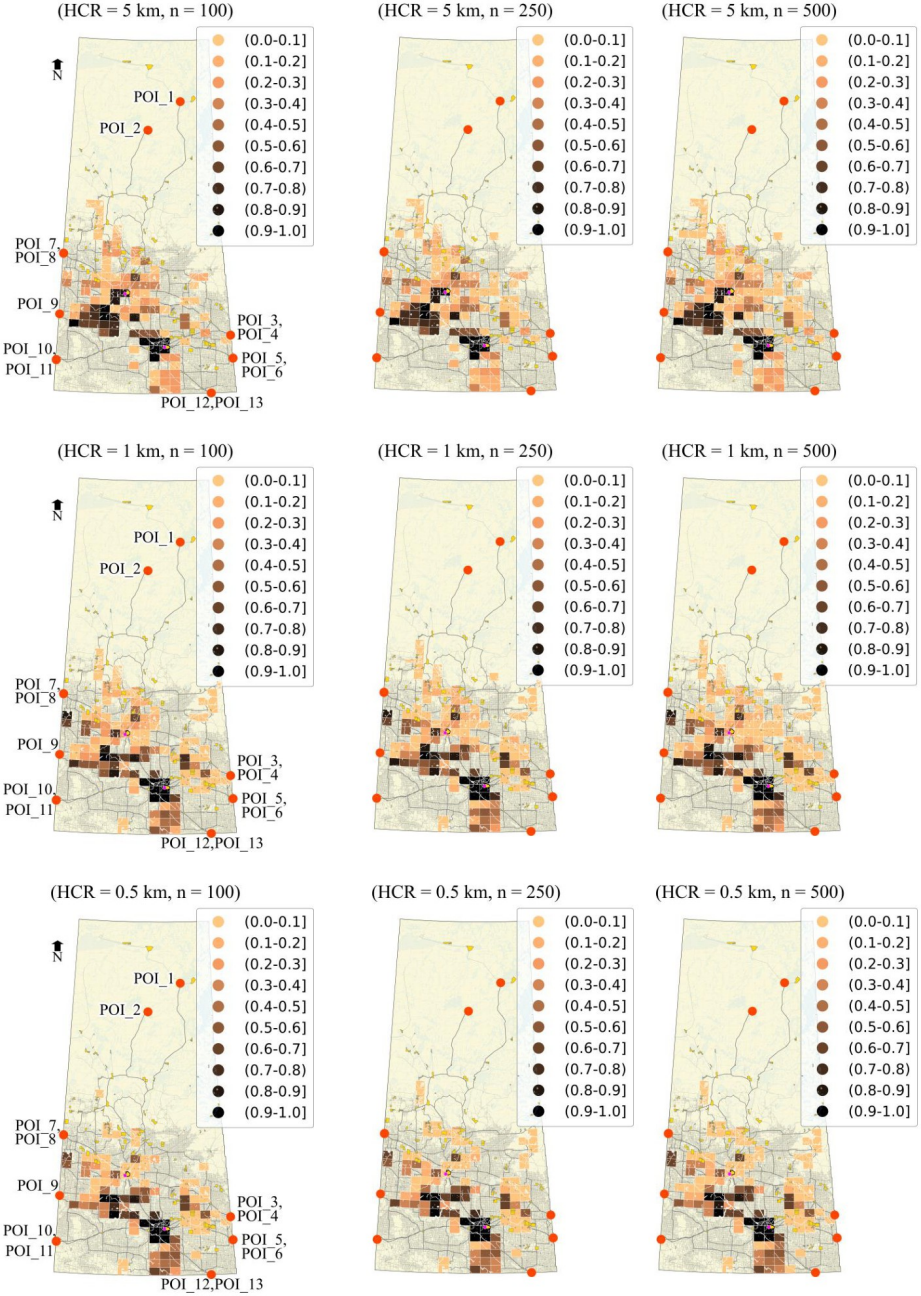
Probability of optimality of zones for case 3 analysis considering fixed hazard circle radius. HCR: Hazard Circle Radius.

It can be noticed that most of the optimal zones (around 84%) that came out from the deterministic analysis also appear as optimal zones in stochastic analysis (with different probabilities). As increasing the iterations to a finitely large number will tend to include every possible risk scenario, the solution guarantees to merge. Note that different accident probabilities on a segment (influential and non-influential reaches) tend to generate risk scenarios in a confined manner which guarantees further to stabilize the number of optimal zones (limited to globally optimal sites) in comparatively a smaller number of iterations. In our demonstration, accident probabilities are assumed uniform throughout the segment reach, which may not be the case always. Fig 19 shows the Pareto optimal zones with fixed HCR of 5, 1 and 0.5 km for 100, 250 and 500 iterations. The same phenomenon can be observed about the increase in the number of zones with more iterations. Also, the number of zones consistently decreases in each case with the reduction of the HCR. However, it is evident from Figs 16, 18 and 19 that certain sites are not appearing as optimal sites no matter how many random iterations we make. In addition, some sites are consistently marked with higher probability of optimality irrespective of the number of iterations and the range or value of hazard circle, which indicates the robustness of the proposed method.

## Conclusion

We propose a methodological framework to evaluate and rank the potential HAZMAT facility sites. The proposed method helps to mitigate the potential risks by evaluating the network of routes between HAZMAT facilities and their associated demand-supply points. It focuses on finding sites that exhibit minimum risk for HAZMAT trips. Although, the demonstration considers the risk to the population and the surrounding water bodies; the safety and security of the transporting packages can be incorporated in the evaluation criteria to seek out the best sites accordingly. We utilized three levels of relationship to develop and quantify a HAZMAT facility’s utility, i.e., local-level, route-level, and zone-level. These hierarchical relationships form the basis for evaluating the sites systematically. Each level offers an opportunity to incorporate different preferences and criteria to develop relationships, which gives a high level of flexibility in HAZMAT transportation risk mitigation decision making. For instance, instead of shortest distance, shortest travel time, least congestion, segment vulnerability or resilience to disruption events, or a composite-cost with different preferences to each criterion can be utilized for developing utilities. At route-level, preference to each of these criteria can be set to optimize the routes, and finally at the zone-level, the importance to POIs can be set to increase the utility weight of the routes from those POIs. The ranking of sites allows decision-makers to choose an optimized facility site in a multi-criterion setting, and stochastic treatment helps to get a different set of optimal sites with an associated probability of optimality. This is useful in aligning the choice with other multidisciplinary criteria to mitigate risk further based on non-transportation-related criteria. It was also attempted to improve the risk functions by incorporating a more comprehensive definition of risk and, at the same time, making use of variables in a relative sense to avoid excessive complexities. We propose a stochastic approach to measure risk, which relaxes some assumptions made in the deterministic analysis. For demonstration purposes, the proposed methods were applied to the Canadian province of Saskatchewan to determine the optimal sites for a potential nuclear facility. Results showed that the stochastic approach captures the variations in risk assessment more realistically and produces a range of optimal sites.

Yet, the variability in decision makers’ preference scores could be considered a new dimension in the analysis. Also, as the analyses were based on the most preferred routes, in the future extensions of this research, the analysis could be expanded to include all feasible routes in the network. This could be done by defining the zone-level utilities based on multiple routes (instead of single routes) between a potential site and the POIs. These multiple routes can be developed based on some pre-defined multi-criteria, by sampling k-shortest routes, by defining routes based on stochastic preference values, or by simply taking the topologically reasonable routes as suggested by [67]. However, this could be a challenging task due to the addition of an additional optimization task at the route-level, provided the limitation of computational resources. These challenges inspire exploring additional methods for data reduction and applications of artificial intelligence and spatial data science methods to reduce the network size and the feasible zones, which will be investigated in future research.

## Supporting information

S1 Fig Determining random incident location based on locational incident probabilities. (TIF)

S1 File Glossary. (DOCX)

S2 File Determining random incident location. (DOCX)

S3 File Data limitations and resources used. (DOCX)

S4 File Pareto-optimality concept. (DOCX)

S5 File The proposed algorithm and model complexity. (DOCX)

S1 Graphical abstract (TIF)
