# Supplementary figures and images for: Hazardous materials facility siting optimization and ranking: A transportation risk mitigation framework
Source: PLoS One. 2023 Nov 15;18(11):e0290723. doi: 10.1371/journal.pone.0290723 (PMC10651046; doi:10.1371/journal.pone.0290723)

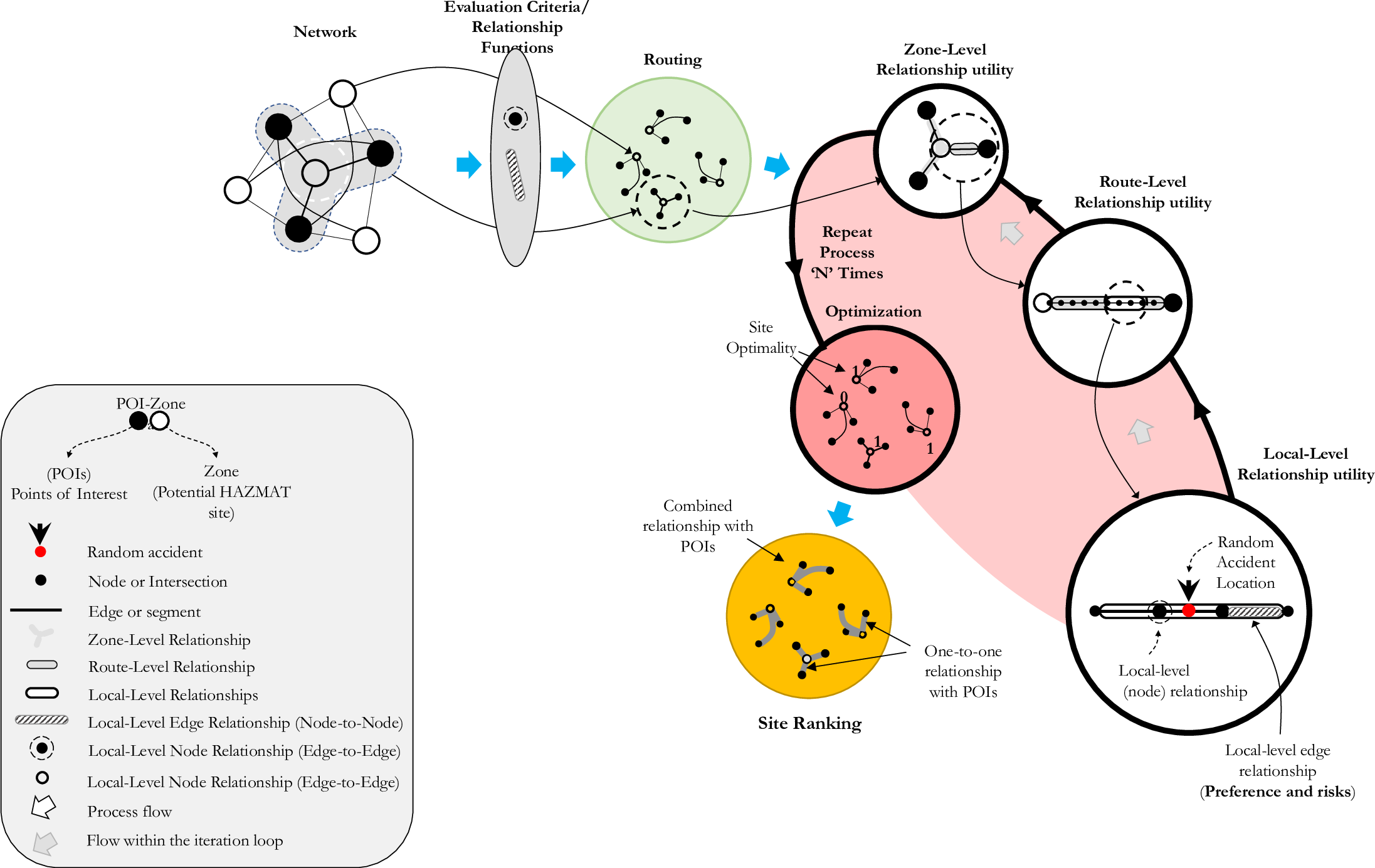

Supplement: S1 Fig — (TIF) [file pone.0290723.s001.tif]
