# Supplementary material for: Hazardous materials facility siting optimization and ranking: A transportation risk mitigation framework
Source: PLoS One. 2023 Nov 15;18(11):e0290723. doi: 10.1371/journal.pone.0290723 (PMC10651046; doi:10.1371/journal.pone.0290723)
Supplement: S1 File — (DOCX) [file pone.0290723.s002.docx]

**S1 Definitions. Glossary.**

| **Term** | **Definitions** |
| --- | --- |
| HAZMAT | Hazardous material |
| OD pair | Origin-destination pair for a route. In the current analysis, it is a pair of a POI and a zone (potential site). |
| Assets | Any valuable entity potentially prone to risk. In current analysis, assets are population and waterbodies. |
| POI(s) | Point of interest(s), such as demand or supply sites. |
| Local element of a network | The smallest network element under consideration, i.e., intersections (nodes) or segments (links or edges) |
| Zone | A potential HAZMAT facility site |
| One-to-one relationship | The relationship between a POI and a zone. |
| Collective relationship | The relationship of a zone with all the POIs combined. |
| Evaluation criteria | Any criteria selected by the decision maker to evaluate the potential sites which expresses the interactive relationship for an OD pair. |
| Route | A route between an origin and a destination (OD pair). In current analysis, the route is between a POI and a zone. |
| Local-level relationship | A relationship at local level (node or edge level) i.e. node-to-node (edge or link) relationship or edge-to-edge (node or intersection) relationship based on an evaluation criterion. |
| Route-level relationship | Relationship exhibits at a route level between a POI and a zone based on an evaluation criterion. |
| Zone-level relationship | Relationship exhibits at a zone level (with all the POIs combined) based on an evaluation criterion. |
| Local-level utility | A quantified value of the relationship utility of local element based on the relationship function considering a criterion. |
| Rout-level utility | A quantified value of the relationship utility at route level based on the local-level utility between a zone and a POI considering a criterion. |
| Zone-level utility | A quantified value of the relationship utility zone level based on the route level utilities between a zone and all POIs combined considering a criterion. |
| Relationship | A desired quality interaction between a POI and a potential facility site or zone. |
| Relationship function | A mathematical expression developed to quantify relationship utility for a local element of the network considering an evaluation criterion. |
| Relationship utility | A quantified value of the quality of relationship based on the relationship functions. |
| Relationship attribute | A relationship function assigned as attribute to a local element of a network. |
| Best route | A path with maximum route-level utility (depending upon the criteria and objective), between a POI and a zone. |
| Shortest route | Best route if the maximum local-level utility for each local element of the route is represented by the minimization of the objective or relationship function. |
| Preferred route | A route developed using the preference of the decision makers (on each segment category in the current analysis) |
| Current analysis | A specific analysis demonstrated in the article within the proposed methodological framework. |
| Segment | A network link between two intersections (nodes) also called as edge. A local element under consideration in the current analysis. |
| Hazard area | The area within which the assets are subject to the risk. |
| Hazard circle | A circular hazard area centered at an incident location. |
| Hazard buffer | A continuous area around the segment with a fixed distance from the segment considered as hazard area. |
| Hazard probability | The probability that a hazardous event will occur. |
| Hazard consequence | The damage created by a hazardous event. |
| Incident probability | The likelihood of happening of an incident. |
| Release probability | The likelihood of the HAZMAT release due to an incident. |
| Consequence probability | The likelihood that the release HAZMAT will pose some consequence. |
| Effective release | The amount of release which have the tendency to pose a negative effect on the surrounding assets. |
| Release and effect probability | The likelihood that a HAZMAT will release and pose an affect on the surrounding assets. |
| Individual incident probabilities | Independent incident probabilities of segments or sections of a segment. |
| Segment incident probability | Incident probability on traversing a segment completely. |
| Locational incident probability | Incident probability at a particular location of on a segment. |
| Deterministic analysis | A risk analysis approach in which hazard area is taken in the form of a fixed distance buffer around the whole segment. |
| Hazard buffer radius | Distance from the segment to the boundary of the hazard buffer. |
| Pareto frontier | A set of non-dominated solutions in Pareto optimality concept. |
| Preferred routes | Routes develop by incorporating decision maker’s preference on network segment categories. |
| Probability of optimality | The probability that a zone will fall on Pareto-frontier |
