# Supplementary material for: Hazardous materials facility siting optimization and ranking: A transportation risk mitigation framework
Source: PLoS One. 2023 Nov 15;18(11):e0290723. doi: 10.1371/journal.pone.0290723 (PMC10651046; doi:10.1371/journal.pone.0290723)
Supplement: S2 File — (DOCX) [file pone.0290723.s003.docx]

# S2 Method. Determining random incident location.

The potential incident's random location can be determined using individual incident probabilities of the segment reaches. For example, suppose a segment is formed by five sections with unique SPFs and incident probabilities, as illustrated in Fig a in S2 Fig. The locational incident probabilities for a particular reach can be calculated using Eqs (10) and (11) and then normalized between [0,1] (Fig b in S2 Fig). A hypothetical scale can then be drawn using the accumulated values of these normalized probabilitie*s* (Fig c in S2 Fig). Note that each section of the scale corresponds to a particular segment section. A random number between [0,1] can be drawn from a uniform distribution onto this hypothetical scale. If, for example, the random number falls between 0.785 and 0.962, the incident is happening in reach $x_{3}$ (Fig d in S2 Fig) and corresponding locational incident probability $P_{x_{3}}$ can be used for risk calculation. In a specific case, when a particular section's calculated locational incident probability comes out zero, that section would automatically be excluded from the scales as an incident cannot happen on that reach. Note that drawing uniformly distributed random numbers on the developed hypothetical scale ensures that the incidents are modelled according to the locational incident probabilities.

S2 Fig. Determining random incident location based on locational incident probabilities.
