# Supplementary material for: Hazardous materials facility siting optimization and ranking: A transportation risk mitigation framework
Source: PLoS One. 2023 Nov 15;18(11):e0290723. doi: 10.1371/journal.pone.0290723 (PMC10651046; doi:10.1371/journal.pone.0290723)
Supplement: S3 File — (DOCX) [file pone.0290723.s004.docx]

# S3 Supporting information. Data limitations and resources used.

**Data limitations and fixtures applied:**

- The Water bodies data used was initially available in two separate feature forms, i.e. polygons (rivers and lakes) and lines (rivers). The risk-based relationships were calculated separately for these two forms. The risk to polygons was calculated by clipping out the affected area (as for population centers); however, the risk to lines was calculated by clipping the affected lengths of the rivers. These two risks were then combined after normalization at the zone level.
- AADT data and the number of lanes for highways were merged from two different sources. The lengths (start and/or endpoints) of the segments in AADT data were not matched with the available network. Therefore, AADT was assigned after a pre-processing of the data, which includes buffering and clipping, etc.
- AADT data for other categories of roads was not available; therefore, synthetic AADT was used for missing segments in light of Highway Functional Classification Concepts, Criteria and Procedures [1]. Values from Table 3-6 (VMT and Mileage Guidelines by Functional Classifications-Collectors and Locals) of [1] were used.
- While applying SPFs, the AADT ranges as recommended by HSM were not considered to ease the demonstration. All one-lane segments were assumed as two-lane rural highways. All three-lane segments were assumed as divided urban highways (Center two-way left-turn lane). All five and six-lane urban segments were considered divided highways (inconsistent data).
- Multiple-vehicle non-driveway collisions were assumed for crashes for SPFs.
- Train movement data and railway network data were two different datasets. Yearly train movement data for railway crossings was available only. This data was converted to billion Train-miles data and applied to the railway segments at crossing only by applying a pre-process. For the rest of the railway segments, the minimum number of Train-miles from calculated Train-miles was used.

**Software used for analyses:**

- All the analysis was done using Python 3.8, including optimization and stochastic processes.
- Geopandas library was used in conjunction with shapely to measure risks. Ref: geopandas.org, shapely.readthedocs.io/en/latest/manual.html
- Networkx library was used for network analysis, including route formation and calculating relationship utilities. Ref: networkx.org/documentation/stable/index.html

# References

1. Federal Highway Administration. Highway Functional Classification Concepts, Criteria and Procedures (2013 Edition). Federal Highway Administration, US Department of Transportation. 2013.
