# Supplementary material for: Hazardous materials facility siting optimization and ranking: A transportation risk mitigation framework
Source: PLoS One. 2023 Nov 15;18(11):e0290723. doi: 10.1371/journal.pone.0290723 (PMC10651046; doi:10.1371/journal.pone.0290723)
Supplement: S4 File — (DOCX) [file pone.0290723.s005.docx]

# S4 Supporting information. Pareto-optimality concept.

**Pareto-optimality**

In Pareto-optimality concept, the non-dominated candidates are sorted. A candidate is non-dominated when there is no better candidate, considering any criterion. Pareto-optimal solutions, in principle, satisfy two conditions; the candidate is, 1) not worse than another candidate considering any of the evaluation criteria; 2) better than other candidates in at least one evaluation criterion. Mathematically, for our current analysis, a candidate zone $z$ is non-dominated if:

|  | $\left( \Omega_{z}^{c} \right)_{\forall c\in C}\leq\left( \Omega_{v}^{c} \right)_{\forall c\in C, \forall v\in Z-\{z\}}$, | S4 (45) |
| --- | --- | --- |
|  | $\left( \Omega_{z}^{c} \right)_{\exists c\in C}<\left( \Omega_{v}^{c} \right)_{\forall c\in C, \forall v\in Z-\{z\}}$, | S4 (46) |

Eq S4 (45) states that the zone-level utility of the candidate zone $z$ should be no worse than other zones $Z-\{z\}$ considering any of the criteria. Eq S4 (46) states that the zone-level utility of the candidate zone z should be better than other zones $Z-\{z\}$ in at least one criterion $(\exists c\in C)$.

**Algorithm to find Pareto-optimal solutions**

The efficient approach to finding the non-dominated solution is presented by [1]. The approach outlined in [1,2] is to sort the population (candidate zones) according to the descending order of importance in the first objective function and rename the population as $P$ of size $N$. Then, set a function $Front(P)$. If $|P|=1$, return $P$ as the output of $Front(P)$, otherwise set top half as $T=Front\left( P^{\left( 1 \right)}-P^{\left( \left| P \right|/2 \right)} \right)$ and the bottom half as $B=Front\left( P^{\left( \left| P \right|/2+1 \right)}-P^{\left( |P| \right)} \right)$. If the $i$-th solution of $B$ is not dominated by any solution of $T$, create a merged set $M=T\cup\{i\}$. Return $M$ as the output of $Front(P)$.

**Complexity**

The complexity of Kung et al.’s approach [1] is $O(N{(logN)}^{|C|-2})$ for $|C|\geq4$ and $O(NlogN)$ for $|C|=2 and 3$, where $N$ is the number of candidate solutions and $|C|$ is the number of evaluation criteria. For large values of $|C|$, the complexity approaches $O(N^{2})$ in the range $100\leq N\leq1000$.

If the objective criteria are increased to large numbers, the algorithm proposed by [3] can be used as it proved to be faster than Kung et al.’s algorithm with a large number of objectives with an overall complexity of $O(|C|N^{2})$ in the worst-case and $O(|C|NlogN)$ in the best case, i.e., when only the top of the summation sequence is the non-dominated solution. Evolutionary Algorithms (EA) heuristics are suitable to solve constrained multi-objective optimization problems of such extent. These algorithms can find multiple solutions in a single iteration. Famous Non-dominated Sorting Genetic Algorithm (NSGA-II) by [4] offers an overall complexity of $O(|C|N^{2})$.

# References

1. Kung HT, Luccio F, Preparata FP. On Finding the Maxima of a Set of Vectors. Journal of the ACM (JACM). 1975;22: 469–476. doi:10.1145/321906.321910

2. Deb K. Multi-objective optimization using evolutionary algorithms. JOHN WILEY & SONS, LTD; 2001. doi:10.1017/S0025557200173498

3. Du J, Cai Z, Chen Y. A sorting based algorithm for finding non-dominated set in multi-objective optimization. Proceedings - Third International Conference on Natural Computation, ICNC 2007. 2007;4: 436–440. doi:10.1109/ICNC.2007.142

4. Deb K, Agrawal S, Pratap A, Meyarivan T. A Fast Elitist Non-Dominated Sorting Genetic Algorithm for Multi-Objective Optimization: NSGA-II. Schoenauer M, Deb K, Rudolph G, Yao X, Lutton E, Merelo JJ, et al., editors. Parallel Problem Solving from Nature PPSN VI. 2000;vol 1917: 849–858. doi:https://doi.org/10.1007/3-540-45356-3_83
