# Supplementary material for: Hazardous materials facility siting optimization and ranking: A transportation risk mitigation framework
Source: PLoS One. 2023 Nov 15;18(11):e0290723. doi: 10.1371/journal.pone.0290723 (PMC10651046; doi:10.1371/journal.pone.0290723)
Supplement: S5 File — (DOCX) [file pone.0290723.s006.docx]

# S5 Algorithm. The proposed algorithm and model complexity.

The proposed model is computationally challenging due to the series of key stages involved in the process. The complexity of the overall model depends on, 1) Calculation of local-level utilities, 2) Determination of preferred routes and respective utilities, 3) Siting optimization, and 4) Finding the probability of optimality.

The complexity of calculating the local-level utilities depends on the formulation of the utility functions, assets located within the network and their size, hazard radius, the size of the network, and the number of route evaluating criteria. The formulation of utility functions determines the total operations to be performed to determine the local-level utility of the network which forms the basis to calculate the best route(s). These operations also depend on the number of spatial components (assets) located around the network. In the demonstrated case, we considered three criteria with local-level utility functions as given in Eqs (22)-(24). Fig 8 shows a linear complexity to calculate these utilities considering the hazard radius of 11Km, with 711 population polygons and 11,593 lake polygons and river lines, i.e.$\boldsymbol{O}({|S|})$, where $|S|$ is the number of segments in the network. With $|C|$ number of criteria, the complexity becomes $\boldsymbol{O}(|C|{|S|})$. The complexity can change with varying number of assets and the hazard radius. The complexity calculated is based on the following proposed algorithm.

| **S5 Algorithm: Calculating local-level utility for population risk** |
| --- |
| Input:  1. Segments of the network $(E)$ with attributes (geometry, accident probability, distance to the nearest emergency service station);  2. Assets (A) with geometries (population pockets or waterbodies);  3. Emergency service stations (ERS) with location coordinates.  Output: Local-level utility for population risk |
| 1 Set $b$ ≔ Hazard radius limit # upper limit on hazard circle radius  2 **For each** edge $e$ in E **do:**  3 Set $p$ ≔ **Get** Individual_segment_probabilities$(e)$  4 Set $l$ ≔ **Get** Accident_location$(e, p)$  5 Set $x$ ≔ **Get** Point_at_accident_location $l$  6 Set $H$ ≔ **Compute** Distance_from_ERS($x,ERS$)  7 Set $X$ ≔ **Get** Point_geometry($x$)  8 Set $B$ ≔ **Get** Random_buffer_within_$b\left( b \right)$ #release/hazard circle radius  9 Set $X$ ≔ **Compute** buffer$(e, B)$  10 Set $A^{'}$ ≔ **Get** All_features_within_$b(A,B)$  11 initialize $Y$≔ [ ]  12 **For each** asset $a$ in $A^{'}$ **do:**  12 Set $A$ ≔ **Compute** Intersection_Area$(a,X)$  13 Set $V$ ≔ Value of vulnerability ratio  14 Set $d$ ≔ **Compute** Nearest_point_between_features$(\cap,X)$  15 Set $D$ ≔ **Get** Population_Density$(a)$  16 Set $y$ ≔ $\frac{\left( A\times D \right)}{d^{2}}\times V$  17 **append** $y$ to $Y$  18 **End**  19 Set ∑ = **Compute** Sum$(Y)$  20 Set $\delta^{\Delta}$ = **Compute** $H\times p\times\sum$  21 **Save** $\delta^{\Delta}$ in $e$ attribute list  22 **End** |

The complexity of calculating the preferred routes and respective route-level and zone-level utilities depends on the number of preferred routes to be considered for each POI and zone, and the total number of POIs and zones considered. As the number of POI-zone pairs increases, the complexity increases. The complexity of Dijkstra’s algorithm for single source shortest paths for a connected network with non-negative weights is $\boldsymbol{O}(\left| S \right|+|X|log|X|)$ [1] and [2], where $|X|$is the number of nodes in the network. With $|O|$ POIs, the complexity is $\boldsymbol{O}\left( \left| O \right|\left( \left| S \right|+\left| X \right|log\left| X \right| \right) \right)$ or $\boldsymbol{O}\left( \left| O \right|\left| S \right|+\left| O \right|\left| X \right|log\left| X \right| \right)$. For $|Q|$ routes for each POI-zone pair, the complexity becomes $\boldsymbol{O}\left( |Q|\left| O \right|\left| S \right|+|Q|\left| O \right|\left| X \right|log\left| X \right| \right)$. If the local-level utilities can be converted to integers, then Dijkstra’s algorithm, as presented in [3] can be implemented in $O\left( |Q|\left| O \right|\left| S \right|+|Q|\left| O \right||X|\left( logWloglogW \right)^{1/3} \right)$, where $W$ is the maximum utility value of a segment in the network.

The complexity for siting optimization varies with the optimization method used. The algorithm by [4] offers worst-case complexity of $\boldsymbol{O}\left( MN^{2} \right)$ when the number of criteria is large (same as NGSA-II algorithm), with $M$ criteria and population size $N$, which in our case is $\boldsymbol{O}(|C|{(\left| C \right|\left| Z \right|)}^{2})$ or $\boldsymbol{O}({|C|}^{3}{|Z|}^{2})$, where $|Z|$ is the number of zones.

The complexity of the overall stochastic model becomes$\boldsymbol{O}\left( |C|{|S|}+\boldsymbol{I}\left( |Q|\left| O \right|\left| S \right|+|Q|\left| O \right|\left| X \right|log\left| X \right|+ {|C|}^{3}{|Z|}^{2} \right) \right)$, where $I$ is the number of iterations (or risk scenarios) considered to calculate the probability of optimality. Note that a slight variation in the complexity can be expected due to the varying number of assets located around the network and the hazard radius considered. Also, variation can be expected based on the methods and algorithms used for stage 2 and 3. Nonetheless, a major part of the complexity depends on the network size $(\left| S \right|$ and$|X|)$ and the number of zones to be evaluated against a given number of POIs. By reducing the network to a manageable size and eliminating impractical zones beforehand, the complexity can be reduced to a large extent.

# References

1. Fredman ML, Tarjan RE. Fibonacci heaps and their uses in improved network optimization algorithms. Journal of the ACM (JACM). 1987;34: 596–615. doi:10.1145/28869.28874

2. Medak J, Pratim P. Review and Analysis of Single-Source Shortest Path Problem Using Dijkstra’ s Algorithm. 2018;20: 10–15. doi:10.9790/0661-2002021015

3. Raman R. Recent results on the single-source shortest paths problem. ACM SIGACT News. 1997;28: 81–87. doi:10.1145/261342.261352

4. Du J, Cai Z, Chen Y. A sorting based algorithm for finding non-dominated set in multi-objective optimization. Proceedings - Third International Conference on Natural Computation, ICNC 2007. 2007;4: 436–440. doi:10.1109/ICNC.2007.142
